# MzmL, a novel marine derived *N*-acyl homoserine lactonase from *Mesoflavibacter zeaxanthinifaciens* that attenuates *Pectobacterium carotovorum* subsp. *carotovorum* virulence

**DOI:** 10.3389/fmicb.2024.1353711

**Published:** 2024-05-09

**Authors:** Lingyun Hao, Jinyou Liang, Shuotian Chen, Junliang Zhang, Yu Zhang, Ying Xu

**Affiliations:** ^1^Center for Plant Environmental Sensing, College of Life Sciences and Oceanography, Shenzhen University, Shenzhen, China; ^2^Shenzhen Key Laboratory of Marine Bioresource and Eco-Environmental Science, Shenzhen Engineering Laboratory for Marine Algal Biotechnology, College of Life Sciences and Oceanography, Shenzhen University, Shenzhen, China

**Keywords:** marine invertebrate, *Mesoflavibacter zeaxanthinifaciens*, quorum quenching, lactonase, MzmL, bacterial soft rot

## Abstract

Quorum sensing (QS) is a conserved cell–cell communication mechanism widely distributed in bacteria, and is oftentimes tightly correlated with pathogen virulence. Quorum quenching enzymes, which interfere with QS through degrading the QS signaling molecules, could attenuate virulence instead of killing the pathogens, and thus are less likely to induce drug resistance. Many Gram-negative bacteria produce *N*-acyl homoserine lactones (AHLs) for interspecies communication. In this study, we isolated and identified a bacterial strain, *Mesoflavibacter zeaxanthinifaciens* XY-85, from an *Onchidium* sp. collected from the intertidal zone of Dapeng Reserve in Shenzhen, China, and found it had strong AHL degradative activity. Whole genome sequencing and blast analysis revealed that XY-85 harbors an AHL lactonase (designated MzmL), which is predicted to have an *N*-terminal signal peptide and share the “HXHXDH” motif with known AHL lactonases belonging to the Metallo-*β*-lactamase superfamily. Phylogenetic studies showed MzmL was closest to marine lactonase cluster members, MomL and Aii20J, instead of the AiiA type lactonases. Ultra performance liquid chromatography-mass spectrometry analysis confirmed that MzmL functions as an AHL lactonase catalyzing AHL degradation through lactone hydrolysis. MzmL could degrade both short- and long-chain AHLs with or without a substitution of oxo-group at the C-3 position, and retained full bioactivity under a wide range of temperatures (28–100°C) and pHs (4–11). Furthermore, MzmL significantly reduced *Pectobacterium carotovorum* subsp. *carotovorum* virulence factor production *in vitro*, such as biofilm formation and plant cell wall degrading enzyme production, and inhibited soft rot development on potato slices. These results demonstrated that MzmL may be a novel type of AHL lactonase with good environmental stability, and has great potential to be developed into a novel biological control agent for bacterial disease management.

## Introduction

Plant pathogenic bacteria is one of the major threats to agricultural production (Sharma et al., 2022). Current disease management strategies for most plant bacterial diseases still rely heavily on pesticide application. Compared to direct killing, strategies that target pathogen virulence factors impose less selection pressure and thus are less likely to induce resistance (Naga et al., 2023). Quorum sensing is a conserved mechanism widely distributed in bacteria, during which each cell produces one or multiple types of signaling molecules, and senses population density through the concentration of these molecules (LaSarre and Federle, 2013). When the signals are accumulated to a certain level above the threshold, the expression of genes involved in different signaling pathways is regulated appropriately so that bacteria adjust their behaviors accordingly to maximize fitness and accomplish host infection. QS is tightly correlated with bacterial virulence, and is involved in regulation of many virulence related behaviors, such as motility, biofilm formation, secretion of extracellular enzymes and production of virulence factors (Jiang et al., 2019). Therefore, disruption of QS through quorum quenching (QQ) may potentially interfere with virulence and result in disease inhibition.

Quorum quenching (QQ) can be achieved through several ways, mainly including interference with the biosynthesis of signaling molecules, signaling molecule degradation, and competition with the signals for specific binding sites (Jiang et al., 2019). Among these, signaling molecule degradation through QQ enzymes, especially acyl-homoserine lactone (AHL) degrading enzymes, has been studied most. AHL is a major group of signaling molecules produced by many Gram negative pathogenic bacteria, which is constituted of a conserved homoserine lactone with an acyl side chain that differs in length and decoration (Valente et al., 2017). To date, more than 50 AHL degrading enzymes have been identified from over 30 bacterial genera including *Bacillus, Chryseobacterium, Enterobacter, Microbacteirum, Agrobacterium, Kurthia, Ochrobactrum, Arthrobacter, Pseudomonas, Solibacillus and Rhodococcus* and so on (Dong et al., 2000; Carlier et al., 2003; Park et al., 2003; Sio et al., 2006; Mei et al., 2010; Wang et al., 2010, 2012; Morohoshi et al., 2012; Dong et al., 2018; Shastry et al., 2018; Ryu et al., 2020). These enzymes can be assigned to three categories, lactonase, acylase and oxidoreductase (Fetzner, 2015). Lactonase inactivates acyl-homoserine lactones by hydrolyzing the lactone ring. This reaction is reversible naturally under acid pH, although it requires a relatively stringent condition, that the open ring product needs 24 h to resume the cyclic structure under a pH less than 2 (Yates et al., 2002). In contrast, the reactions catalyzed by acylase and oxidoreductase are irreversible (Torres et al., 2017). The *Escherichia coli* or *Pectobacterium* transformants expressing QQ enzymes either significantly inhibited soft rot development or had much lower virulence in *ex vivo* plant tissues, such as potato, cucumber and carrot slices, and cabbage leaves (Garge and Nerurkar, 2016). Furthermore, transgenic tobacco and tomato plants expressing the QQ enzymes showed higher resistance to *Pectobacterium* infection (Fray, 2002). These findings supported the application potential of the QQ approach in bacterial disease management. Further exploitation of novel QQ enzymes with attractive physical and chemical properties such as good environmental stability and high enzymatic efficiency, and investigation of appropriate ways to ensure stable expression of these enzymes *in planta* is critical to facilitate their real-world application.

In the past decades, marine bacteria have been increasingly recognized as a rich bioresource of novel bioactive compounds and enzymes (Torres et al., 2019). Due to the extreme variation in marine environmental factors, such as temperature, salinity, and osmotic stress, these bacteria have evolved unique metabolic pathways to produce various bioactive compounds to help them survive in particular niches, compete with other microbes, and facilitate their hosts to maximize fitness. As a result, marine microbes are capable of producing extremely diversified compounds with various structures and bioactivities, and the occurrence of certain bioactive substances has been shown to exceed their terrestrial counterparts. For example, it was reported that the percentage of marine bacteria with quorum quenching bioactivities was much higher than that of agricultural soil or crop rhizosphere (Torres et al., 2019). Therefore, it is highly likely to discover novel QQ enzymes from marine bacteria. To date, several novel QQ enzymes with unique bioactivities and biochemical properties from various marine sources, such as sediments, fish mucus, healthy shrimp and breams, have been identified and characterized (Romero et al., 2011; Huang et al., 2012; Morohoshi et al., 2015; Tang et al., 2015; Cai et al., 2018). However, few reports were seen from marine invertebrates associated bacteria. Furthermore, the application of marine derived QQ enzymes to combat plant bacterial disease remains understudied.

In this study, a bacterial isolate XY-85 from a marine invertebrate sample, identified as *Mesoflavibacter zeaxanthinifaciens*, was found to have QQ bioactivity. Initial High-performance liquid chromatography (HPLC) analysis and the acidification assay suggested the bioactive substance produced by XY-85 might be a lactonase. The aim of this study is to identify and characterize the lactonase encoding gene from XY-85, and examine the physiochemical properties of the purified enzyme. The effects of XY-85 lactonase on *Pectobacterium carotovorum* subsp. *carotovorum* (*Pcc*) virulence factor production as well as soft rot development were also examined to determine the potential of this lactonase to be developed into a biological control agent in plant bacterial disease management.

## Materials and methods

### Bacterial isolation

A marine invertebrate sample, *Onchidium* sp., collected from the attached stone at the intertidal zone in Dapeng Reserve in Shenzhen (Guangdong Province, China) was used for bacterial isolation. The sample was fully ground in liquid nitrogen and suspended in 10 times of sterile sea water (17 g/L, weight: volume). The entire homogenates were evenly spread onto the R2A plates (R2A; BD Difco) supplemented with sea salt (17 g/L), with a 100 μL aliquot per plate. The plates were incubated at room temperature (25°C) for a week. Colonies on the plates were observed with a dissecting microscope each day. Morphologically distinct single colonies were selected, subcultured onto the Marine Agar (MA; BD Difco) plate, and purified twice with streak plating. Then purified colonies were suspended into 5 mL of sterile glycerol stock [75% Marine Broth 2216 and 25% glycerol (v/v)] and stored at −80°C in four replicates.

### Bacterial strains, media, growth conditions, and chemicals

XY-85 was cultured in marine broth 2,216 (MB; BD Difo^™^) at 28°C. *E. coli* strains DH5α and BL21 (DE3) were cultured on Luria–Bertani (LB) agar at 37°C and used as hosts for expressing the protein whose encoding gene was cloned into pET-28a (Ke Lei Biotechnology Co., Ltd). The AHL biosensors *Chromobacterium violaceum* CV026 and VIR24 (McClean et al., 1997; Someya et al., 2009), used to detect short-chain (C_4_ to C_8_) and long-chain (C_8_ to C_14_) AHLs, were maintained on LB agar at 28°C. *Pectobacterium caratovorum* subsp. *caratovorum* (*Pcc*, provided by Dr. Junna He at China Agricultural University) was cultured on Luria–Bertani (LB) agar at 28°C. Kanamycin was added at 50 μg mL^−1^. C_4_-HSL, C_6_-HSL, 3-oxo-C_6_-HSL, and C_8_-HSL were purchased from Cayman Chemical Company (Ann Arbor, MI, United States); 3-oxo-C_8_-HSL, C_10_-HSL, 3-oxo-C_10_-HSL, C_12_-HSL, 3-oxo-C_12_-HSL, C_14_-HSL, and 3-oxo-C_14_-HSL were purchased from Sigma-Aldrich (St. Louis, MO, United States). All of the AHL stock solutions (10 to 500 mM) were prepared in dimethyl sulfoxide (DMSO) and stored under −20°C.

### Bacterial identification

Bacterial DNA was extracted with the FASTDNA Spin Kit for Soil (MPBIO, United States) following the manufacturer’s instructions. The 16s rDNA region was amplified via PCR, using the forward and reverse primers, 1492R and 27F, as previously described (Lane, 1991). The purified PCR product was sequenced at Beijing Genomics Institute. Species identification was conducted using the blastn program,^1^ based on alignment of the sequencing results with existing 16s rDNA sequences in the “Nucleotide collection (nt/nt)” database using Megablast. The first ranked strain with full genus and species names and >98.5% similarity to the query was selected as the closest match. Sequences of the 16s rDNA region of all isolates had been deposited to the GenBank database with the accession No. OR262693-OR262889.

### Screening of bacteria with QQ activity

Screening for bacterial isolates with QQ activity was done as previously described with some modification (Su et al., 2019). Briefly, each strain was individually streaked onto a MA plate and incubated at 28°C for 48–72 h. Single colonies were then picked and inoculated into 10 mL SGTYP medium and incubated at 28°C with shaking (200 rpm). After 3 days, a 900 μL aliquot of each bacterial culture was mixed with 100 μL, 1 M PIPES buffer (Tang et al., 2013) with 1 μL of 10 mM C_6_-HSL (Sigma) supplemented, and the total mixture was added into a well of a 24-well plate. Plates were shaken at 28°C, 200 rpm for 24 h. Negative controls were those with 900 μL SGTYP medium only. Then cultures were collected and centrifuged at 13,500 rpm for 5 min, and 10 μL supernatant of each culture was added to a 90 μL overnight culture of CV026 grown in LB broth. After incubation at 28°C with shaking at 200 rpm for 12 h, the cultures were centrifuged at 13,500 rpm for 5 min and the supernatants were discarded. One hundred microliters of DMSO was added to the pellet and vortexed vigorously to extract violacein. After centrifugation at 13,500 rpm for 5 min, the optical density of the supernatants was measured at 550 nm (OD_550_). In addition, to confirm that the QQ bioactive isolates did not produce antimicrobial compounds towards CV026, the cell density of CV026 was determined by OD_595。_ Four replicate wells were included for each treatment. The experiment was repeated at least three times.

### Initial HPLC analysis

Initial analysis of the potential QQ bioactive compound in XY-85 fermentation culture was performed with HPLC. Briefly, 4 mM C_6_-HSL was incubated with 1 mL of XY-85 3-day-old fermentation culture, and a 200 μL aliquot was sampled at 0, 8, and 14 h, respectively, and extracted by acetyl acetate. The extracts were analyzed through a HPLC system (Shimadzu, Japan) with Luna C_18_ (2) reverse-phase column according to Wang et al. (2010).

### Acidification assay

To determine if the QQ compound in 85 fermentation culture is a lactonase, an acidification assay was conducted as described by Uroz and Heinonsalo (2008) with some modifications. Briefly, 10 μM C_6_-HSL was incubated with 900 μL of 3-day-old XY-85 fermentation culture and 100 μL of 1 M PIPES for 24 h. Then cells were discarded by centrifugation at 15,000 rpm for 2 min and the supernatant was acidified with HCl to pH 1 and incubated at 4°C for 24 h. Bioactivities of the acidified supernatants to induce CV026 pigment production were examined as described above in the screening assay. Equal amounts of C_6_-HSL first incubated with SGTYP medium and 10 mM NaOH, respectively, at room temperature for 30 min, and then acidified with HCl were used as negative and positive controls. Two replicates were included for each treatment and the experiment was repeated three times.

### Identification and phylogenetic analysis of a lactonase-encoding gene, GM002175, in XY-85

To identify the lactonase-encoding gene in XY-85, whole genome sequencing of the bacterium was conducted. Briefly, XY-85 was cultured on MA plates at 28°C for 3 days and bacterial genomic DNA was extracted using the FastDNA^®^ SPIN Kit for Soil kit (MP BIO, United States) and sent for sequencing. A total amount of 1 μg DNA per sample was used as input material for the DNA sample preparations. Sequencing libraries were generated using NEBNext^®^ Ultra™ DNA Library Prep Kit for Illumina (NEB, United States) following manufacturer’s recommendations and index codes were added to attribute sequences to each sample. Briefly, the DNA sample was fragmented by sonication to a size of 350 bp, then DNA fragments were end-polished, A-tailed, and ligated with the full-length adaptor for Illumina sequencing with further PCR amplification. At last, PCR products were purified (AMPure XP system) and libraries were analyzed for size distribution by Agilent2100 Bioanalyzer and quantified using real-time PCR. The whole genome of XY-85 was sequenced using Illumina NovaSeq PE150 at the Beijing Novogene Bioinformatics Technology Co., Ltd. The raw data was filtered to obtain clean data, which then was assembled by the SOAP denovo, SPAdes, Abyss, CISA and gapclose software. Fragments below 500 bp were filtered out and the final result was counted for gene prediction.

Long interspersed repeated sequences and tandem repeats were annotated and identified by RepeatMasker (version 4.0.5) and Tandem repeats finder (version 4.07b), respectively. The coding genes were annotated using GeneMarkS (version 4.17) with defaulted parameters. Genome component prediction included the prediction of the coding gene, repetitive sequences, non-coding RNA, genomics islands, transposon, prophage, and clustered regularly interspaced short palindromic repeat sequences (CRISPR) using the following databases: GO (Gene Ontology), KEGG (Kyoto Encyclopedia of Genes and Genomes), COG (Clusters of Orthologous Groups), NR (Non-Redundant Protein Database), TCDB (Transporter Classification Database), and Swiss-Prot. A whole genome Blast search (*E*-value less than 1×10^−5^, minimal alignment length percentage larger than 40%) was performed against above-described databases. Putative QQ enzyme-encoding genes were searched from whole-genome sequencing data through local BLASTP against known QQ enzymes in the above described databases.

Phylogenetic analysis was conducted with the MEGA-X program with neighbor-joining method (1,000 bootstrap replicates) to show the phylogenetic relationship among MzmL and the previously reported QQ enzymes. The nucleotide sequence of *mzmL* gene from XY-85 has been deposited in the GenBank database under accession No. PP179376.

### Heterologous expression of GM002175 and protein purification

Cloning of the lactonase-encoding gene, GM002175, was carried out as previous described (Sambrook et al., 1989). Full length of GM002175 was amplified with primers 85-NH-For (5′-ATACC ATGGGCCATCATCATCATCATCACAAAAAACTAATCATTCTT CTTATTACC-3′) and 85-NH-Rev (5′-CGCCTCGAGCTATTGAA TTGGTTCTGGTG-3′). After double digestion with NcoI and XhoI at 37°C for 1 h, the fragment was cloned into the pET28a vector digested with the same endonucleases. The recombinant vector was transformed into DH5α by electroporation. After recovery in LB medium at 37°C for 1 h, the culture was spread onto LB plate plus 50 μg/mL kanamycin. After verification through PCR using primers pET-For: (5′-CGTCGTCGGTTGAGTCGAAGG-3′) and pET-Rev: (5′-TACGCAGGCCGCATCTCCTAG-3′), the positive clone was transferred to LB medium plus kanamycin and shaken at 37°C overnight. The recombinant plasmid was extracted with TIANprep Mini Plasmid Kit and then transformed into *E. coli* BL21 (DE3) competent cells.

Protein purification was carried out as described by Tang et al. (2015) with some modification. Briefly, the positive transformant was inoculated to fresh LB medium plus kanamycin and shaken at 37°C for 12 h, and then 1:20 (vol:vol) transferred into fresh LB medium plus kanamycin. After 2 h incubation at 37°C, isopropyl-β-D-thiogalactopyranoside (IPTG) was added to a final concentration of 0.1 mM, and induced at 18°C overnight. The cells were pelleted by centrifuging at 15,000 rpm, 4°C for 5 min. The pellet was re-suspended in balance buffer (200 mM NaCl, 50 mM Tris, pH = 8.0) and lysed using an Ultrahigh Pressure Homogenizer at 4°C, 800 bar for 5 min. The mixture was centrifuged at 15,000 rpm, 4°C for 30 min, and the supernatant was filtrated with 0.22 μm filter. The protein was purified by NGC chromatography system with an Ni-NTA affinity chromatography column (bio-rad). After loading the filtered sample, the column was washed with balance buffer, 10, 20 and 60 mM imidazole sequentially, and eluted with 500 mM imidazole. The purified proteins were analyzed with SDS-PAGE and stored at −20°C in 25% glycerol.

### Ultra performance liquid chromatography-mass spectrometry analysis of products formed by MzmL catalysis

Confirmation of the degradation product of the purified XY-85-lactonase, MzmL, was conducted by ultra performance liquid chromatography-mass spectrometry (UPLC-MS). Five hundred microliters of the reaction mixture containing 1 mM C_6_-HSL and 0.3 μg/mL purified protein were incubated at 28°C. Samples were taken at 0 and 24 h incubation, and an equal volume of methanol was added to stop the reaction and then centrifuged at 12,000 rpm for 20 min. The supernatant was analyzed by UPLC using a ACQUITY UPLC^®^ BEH C18 reversed-phase column (1.7 μM; 4.6 by 100 mm) with a mobile phase of acetonitrile-water [0.1% formic acid; a linear gradient (vol/vol) of acetonitrile from 5 to 95% over 10 min at a flow rate of 0.3 mL min^−1^ for C_6_-HSL]. The separated fractions were further analyzed by electrospray ionization mass spectrometry (ESI-MS).

### Determination of the degradative activities of MzmL for various AHL molecules

To determine the degradation spectrum of MzmL, a 1 mL mixture containing 3 μM enzyme, 20 μM AHL and 980 μL PIPES buffer was incubated at 28°C for 24 h. Negative controls were those with 18 μL ddH_2_O only. Then 10 μL of each culture was added to a 90 μL overnight culture of CV026 (for the short chain AHLs, C_6_-HSL and 3-oxo-C_6_-HSL only) or VIR24 (for the rest of AHLs) grown in LB broth. Violacein production was quantified as described above. Four replicate wells were included for each treatment. The experiment was repeated at least three times.

To determine the kinetic activities of MzmL, a 100 μL mixture containing 5 nM enzyme, 100 μM BTB, 2.5 mM pholinepropanesulfonic acid (MOPS) buffer (pH 7.1), and 0 to 2.5 mM substrate was incubated at 25°C, respectively. After 3 min, the color change was measured OD_630_. A standard curve representing the relationship between the absorption change and proton concentration was generated with HCl. The K_m_ values were calculated with GraphPad Prism software (version 7) with the Michaelis–Menten equation. Four replicates were included for each treatment and the experiment was repeated at least twice.

### Residual bioactivity of MzmL upon temperature, pH and metal ion treatments

Relative bioactivity of MzmL at different temperatures and pH was determined as described previously (Tang et al., 2015). Briefly, purified MzmL protein was preincubated at 28, 40, 60, 80, and 100°C, respectively, for 30 min, or at different pHs (2–12, interval = 1) at 4°C for 3 h, after which the residual activity to degrade C_6_-HSL was evaluated in the same manner as described above. Two replicates were included for each treatment and the experiment was repeated twice.

The sensitivity of MzmL to metal ions was detected as described by Tang et al. (2015) with some modification. Briefly, 5.7 μg of purified MzmL protein dissolved in 2.5 mM of MOPS buffer (pH 7.1) was incubated with 0.1 M of different metal ions (Ni^2+^, Mg^2+^, Mn^2+^, Ca^2+^, Cu^2+^, Zn^2+^, Co^2+^, and Fe^2+^), or with 1 mM EDTA at 4°C for 1 h. Then, the EDTA-treated MzmL protein was re-incubated with 1.1 mM of metal ions. To measure the residual activity of MzmL protein, 87.5 μL of protein, 10 μL of 1 mM of BTB solution and 2.5 μL of 40 mM of C_6_-HSL were added to 96-well plate and the decline of OD_630_ was measured in 2 min. For each assay, two replicates were performed and the experiment was repeated for three times.

### Degradation of *Pcc*-AHL by XY-85 fermentation culture

Extraction of the AHL signals produced by *Pcc* was done as previously described (Li et al., 2022). Briefly, *Pcc* was grown in 100 mL of LB medium at 28°C with shaking (200 rpm) for 24 h. The culture was centrifuged at 15,000 rpm for 5 min, and ethyl acetate, three times of the cultural volume was added to extract the AHL in the supernatant. The crude extract was prepared as a 50 mg/mL stock in DMSO. Nine hundred microliters of the 3-day-old XY-85 fermentation culture and 100 μL, 1 M PIPES solution were co-incubated with the *Pcc*-AHL crude extract, which was supplemented at a final concentration of 100 μg/mL for 24 h, before detection with CV026 as described above. Equal amount of *Pcc*-AHL only was included as positive controls. Two replicates were included for each treatment and the experiment was repeated three times.

### Impacts of MzmL on *Pcc* virulence factor production *in vitro*

The impacts of MzmL on extracellular plant cell wall degrading enzyme production in *Pcc* were examined as previously described with some modifications (Joko et al., 2014). Briefly, medium plates for detection of pectate lyase (Pel), polygalacturonase (Peh) and cellulase (Cel) production were prepared as described by Joko et al. (2014), and the plates for detection of protease (Prts) were prepared with 0.8% agarose plus 1% skim milk, with four holes made per plate at equidistant distance from each other by a sterile glass tube (7 mm in diameter). *Pcc* was cultured in 1 mL of LB broth supplemented with 100 μg/mL MzmL for 0, 12, 24 h. The cultures were centrifuged at 15,000 rpm for 5 min, and supernatants were filtrated with 0.22 μm filters. Then a 20 μL aliquot of the culture was added into each hole. Cells grown in LB alone were used as negative controls. All plates were sealed with parafilm and incubated at 28°C for one (for Pel, Peh and Cel) or 3 days (for Prts). The Pel and Peh plates were developed with 5 M H_2_SO_4_, while Cel plates were developed with 1% Congo red solution for 15 min. The Cel plates were washed with 1 M NaCl solution for 5 times and 10 min for each time. Diameters of halos developed surrounding the inoculation sites were measured. Inhibition percentages of MzmL on each enzyme production was calculated as: % inhibition = 100% × (diameter of control − diameter of treatment)/(diameter of control). Four replicates were included for each treatment and the experiment was repeated three times.

*Pcc* cells grown overnight in YP medium (peptone 10 g/L, yeast extract 5 g/L, pH = 6.8) at 28°C were adjusted to 1 × 10^7^ CFU/mL with SOBG medium (tryptone 20 g/L, yeast extract 5 g/L, NaCl 0.5 g/L, KCl 0.186 g/L, MgSO_4_ 7H_2_O 9 mM, glycerol 20 g/L, pH = 7.0), and MzmL was supplemented to a final concentration of 5 μg/mL. The bacterial suspension supplemented with an equal volume of glycerol was used as negative control. A 2 mL mixture of each treatment was aliquoted to a sterile glass tube, and incubated at 28°C without shaking. After 4 days, biofilm produced in each tube was examined using the crystal violet staining method as described by previously (Haque et al., 2017).

### Inhibition of soft rot development by *mzmL* in potato slices

*Pcc* cells grown overnight in LB broth was collected and washed twice with sterile ddH_2_O, and resuspended in ddH_2_O to adjust to a cell density of 1 × 10^5^ CFU/mL. Recombinant *E. coli* (DE3) cells carrying pET28a and pET28a::*N6his*:*mzmL* were harvested after IPTG induction at 18°C overnight respectively, and resuspended in ddH_2_O to adjust to 1 × 10^10^ CFU/mL. Potatoes purchased from a local supermarket were surface sterilized with 1% sodium hypochlorite first and then 75% ethanol for 30 s for each step, dried under room temperature and cut into slices of ~7 mm in thickness. Equal volumes of *Pcc* and *E. coli* cell suspensions were mixed together, and a 5 μL bacterial mixture was inoculated to the center of each potato slice. All slices were placed in sterile peri dishes sealed in plastic bags, and incubated at 28°C for 48 h. Watery rotten lesions around inoculation sites were observed as disease symptoms, and the lesion areas were pictured and quantified through Image J (Su et al., 2019). Approximately 20 replicates were included in each treatment and the experiment was repeated three times.

### Statistical analysis

All statistical analyses were performed using the R program (version 4.3.1; R Development Core Team, R Foundation for Statistical Computing; http://www.R-project.org). Tukey’s honestly significant difference test (based on one-way analysis of variance) was used to compare the differences in the mean values among treatments for the acidification and environmental stability assays. Students’ *t*-test was used to compare differences between the mean values of each compound treatment and control group of the QQ bioactivity screening assay, degradative spectrum assay, virulence factor production assay and the virulence assay. Significance was set at a *p* < 0.05 for all aforementioned tests.

## Results

### Isolation and identification of a *Mesoflavibacter zeaxanthinifaciens* isolate XY-85 capable of degrading C_6_-HSL

**Figure 1:**
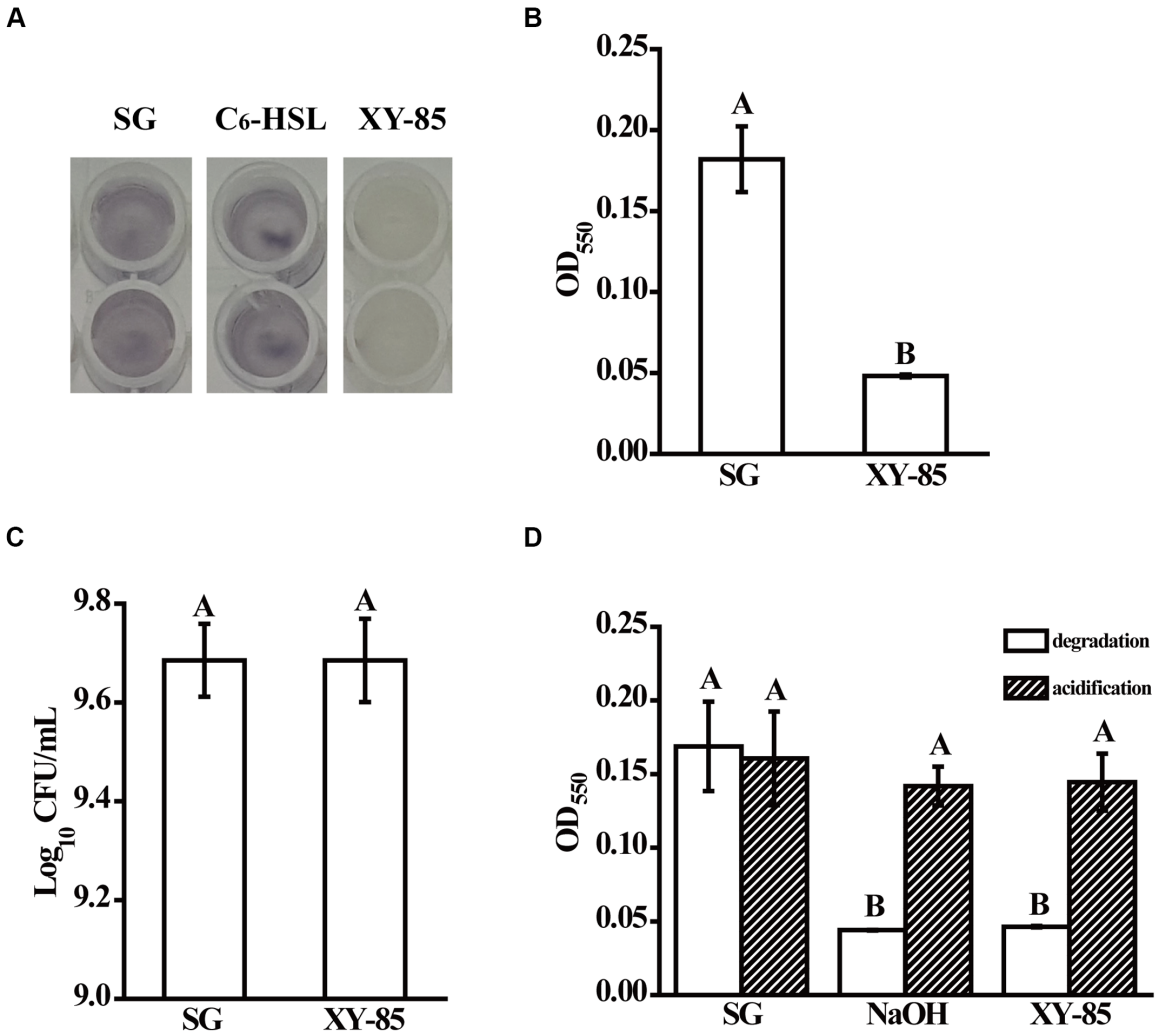
Quorum quenching bioactivity of *Mesoflavibacter zeaxanthinifaciens* XY-85. Shown are **(A)** cultural color change, and **(B)** Mean violacein production of *Chromobacterium violaceum* CV026 after overnight incubation with the reaction supernatants from different groups, **(C)** mean populations of CV026 after culturing with the reaction supernatants from different groups, and **(D)** mean violacein production of CV026 after overnight incubation with reaction supernatants from C_6_-HSL pre-treated in different groups (degradation), and C_6_-HSL pre-treated in different groups first and then acidified with 1 M HCl for 24 h (acidification), respectively. SG: C_6_-HSL pre-incubated with SGTPY medium only for 24 h; XY-85: C_6_-HSL pre-incubated with XY-85 culture for 24 h; C_6_-HSL: C_6_-HSL only. The mean values were calculated based on twelve **(A–C)** and six replicates **(D)** from three independent experiments, respectively, and the bars were standard errors. Different letters represent a significant difference of *p* < 0.05 by student’s *t*-test **(A–C)**, and Tukey’s honest significant difference test **(D)**, respectively.

A total of 197 bacterial strains were isolated and identified from an *Onchidium* sp., an invertebrate collected from Dapeng Bay at Shenzhen. These bacteria were categorized into 53 genera based on 16s rRNA sequencing (Table 1; Supplementary Table S1). *Bacillus*, *Sulfitobacter* and *Ruegeria* were the top three abundant genera, altogether representing ~37.6% of the sample pool, followed by *Paracoccus, Pseudovibrio, Vibrio, Tanacibaculum, Mesoflavibacter, Alteromonas and Erythrobacter* sp., which contained 5–11 isolates, respectively. For the rest of the genera, less than 5 isolates were present in each of them. Among these, a total of 32 isolates from the genus *Acinetobacter, Bacillus, Erythrobacter, Mesoflavibacter, Microbacterium, Neptunomonas, Paracoccus, Pararhodobacter, Pseudooceanicola, Pseudoreugeria, Rhodococcus, Roseomonas, Sphingorhabdus*, and *Tenacibaculum* showed C_6_-HSL degrading capability *in vitro* using a reporter assay, which accounted for ~16.24% of the total isolates (Supplementary Figure S1A and Supplementary Table S2). Furthermore, endpoint OD readings of CV026 confirmed that these marine bacterial cultures did not produce antimicrobial compounds under the tested conditions (Supplementary Figure S1B). One of the isolates, *Mesoflavibacter zeaxanthinifaciens* XY-85, showed C_6_-HSL degrading bioactivity without having negative impacts on CV026 growth (Figures 1A–C). Since no QQ enzyme has been purified from bacteria of this genus before, XY-85 was chosen for further investigation.

**Table 1:** Summary of classified bacterial isolates with or without quorum quenching (QQ) bioactivities associated with an *Onchidium* sp. collected from Dapeng Bay at Shenzhen, China.

	Genus	No. of isolates	Strain number	QQ bioactivity
	***Acinetobacter***	1	XY-205	√
		1	XY-91	×
	*Ahrensia*	2	XY-113, 222	×
	*Algibacter*	1	XY-114	×
	*Alteromonas*	3	XY-153, 165, 170	×
	*Alteromonas*	4	XY-150, 169, 174, 187	×
	*Antarctobacter*	1	XY-81	×
	*Aquimarina*	1	XY-208	×
	***Bacillus***	6	XY-20-2, 24-2, 79, 93, 98, 379	√
		27	XY-25, 32, 35, 37, 40, 41, 43, 46, 48, 51, 55, 58, 63, 65, 68, 69, 95, 102, 106, 111, 128, 186, 191, 199, 224, 228, 362	×
	*Brevibacterium*	2	XY-216, 256	×
	*Brevundimonas*	3	XY-252, 364, 369	×
	*Cellulophaga*	3	XY-154, 157, 158	×
	*Cellvibrio*	1	XY-145	×
	*Donghicola*	1	XY-67	×
	*Echinicola*	1	XY-200	×
	***Erythrobacter***	5	XY-185, 207-2, 230, 238, 336	√
	*Fangia*	1	XY-144	×
	*Flavobacterium*	1	XY-259	×
	*Glutamicibacter*	2	XY-162, 184	×
	*Labrenzia*	2	XY-192, 355-2	×
	*Lacinutrix*	4	XY-73, 83, 112, 116	×
	*Leisingera*	1	XY-116	×
	*Leucobacter*	2	XY-143-2, 382	×
	*Loktanella*	1	XY-334-1	×
	*Luteimonas*	3	XY-21, 131, 345	×
	*Lysobacter*	1	XY-343	×
	*Maribacter*	3	XY-87, 110, 176, 325	×
	*Marinomonas*	1	XY-23	×
	***Mesoflavibacter***	7	XY-85, 108, 122, 135, 136, 166-2, 221	√
	***Microbacterium***	3	XY-151, 203, 209	√
	*Microbulbifer*	1	XY-92	×
	***Neptunomonas***	1	XY-337	√
	*Nonlabens*	4	XY-118, 172-1, 175, 179	×
	*Ochrobactrum*	1	XY-130	×
	***Paracoccus***	1	XY-322	√
		10	XY-20-1, 30, 34, 146, 148, 167, 172-2, 180, 182, 330	×
	***Pararhodobacter***	1	XY-270	√
	*Phaeobacter*	1	XY-126-1	×
	*Pseudoalteromonas*	1	XY-133	×
	***Pseudooceanicola***	1	XY-99	√
	***Pseudoruegeria***	1	XY-210-2	√
		1	XY-164-2	×
	*Pseudovibrio*	9	XY-45-1, 49, 50, 53, 59, 90, 96, 104, 107	×
	***Rhodococcus***	1	XY-127	√
	***Roseomonas***	1	XY-339	√
	*Roseovarius*	1	XY-28	×
	*Ruegeria*	20	XY-27, 84, 86, 101, 109, 137, 142, 152, 159, 181-2, 189, 220-1, 247, 300, 319, 321, 323, 328, 361, 363	×
	*Sagittula*	1	XY-166-1	×
	*Salinimonas*	1	XY-15-2	×
	*Shimia*	2	XY-123, 333	×
	***Sphingorhabdus***	1	XY-355	√
	*Sulfitobacter*	21	XY-66, 71, 72, 78, 89, 115, 119, 125-2, 164-1, 173-1, 177, 181-1, 188, 190, 197, 202, 204, 206, 207, 269, 331	×
	***Tenacibaculum***	2	XY-163, 178	√
		6	XY-29, 56, 117, 156, 160, 171	×
	*Thalassococcus*	1	XY-70-1	×
	*Tropicibacter*	1	XY-155	×
	*Vibrio*	8	XY-26, 39, 47, 54, 94, 134, 140, 367	×
	*Zobellia*	2	XY-121, 193	×
Total No.	54	197		32

Shown is the classification and counting of all bacterial strains with or without QQ bioactivity associated with an *Onchidium* sp. Classification was conducted based on comparison of bacterial 16s ribosomal DNA sequences with those in the NCBI database. The numbers of isolates assigned to each genus were counted. QQ bioactivity was detected by using the bioreporter strain *Chromobacterium violaceum* CV026, and the genera cotaining QQ bioactive strains are highlighted in black.

### HPLC analysis and acidification assay suggested that XY-85 produced a lactonase

To confirm whether XY-85 produced a QQ enzyme, supernatants of C_6_-HSL incubated with the XY-85 culture at different time points were collected and analyzed by HPLC. A single sharp peak was observed at 0 h, indicating the presence of the intact compound C_6_-HSL. After 14 h incubation with the XY-85 fermentation culture, the peak completely disappeared, indicating that the QS inhibitor produced by XY-85 was likely to be a QQ enzyme (Supplementary Figure S2).

Known AHL degrading enzymes include lactonase, acylase, and oxidoreductase (Dessaux et al., 2011). Among these, lactonase degrades AHL in a reversible manner, that the inactivated product with an open homoserine lactone ring can be re-circularized under acid conditions and thus restore its bioactivity to induce quorum sensing (Uroz and Heinonsalo, 2008). To determine which kind of enzyme XY-85 produced, an acidification assay was performed. As expected, C_6_-HSL was degraded by 10 mM NaOH and thus was not able to induce violacein production in CV026. Upon acidification, violacein production was observed suggesting the circular structure and bioactivity of C_6_-HSL was resumed (Figure 1D). Similar finding was observed with the XY-85 treatment, that C_6_-HSL incubated with XY-85 culture was degraded but resumed bioactivity under acid condition. These results suggested that the QQ enzyme produced by XY-85 was likely to be a lactonase, which is known to hydrolyze AHL to generate an open-ring compound and can self-circularize under acid pH.

### Identification of a lactonase encoding gene in XY-85

**Figure 2:**
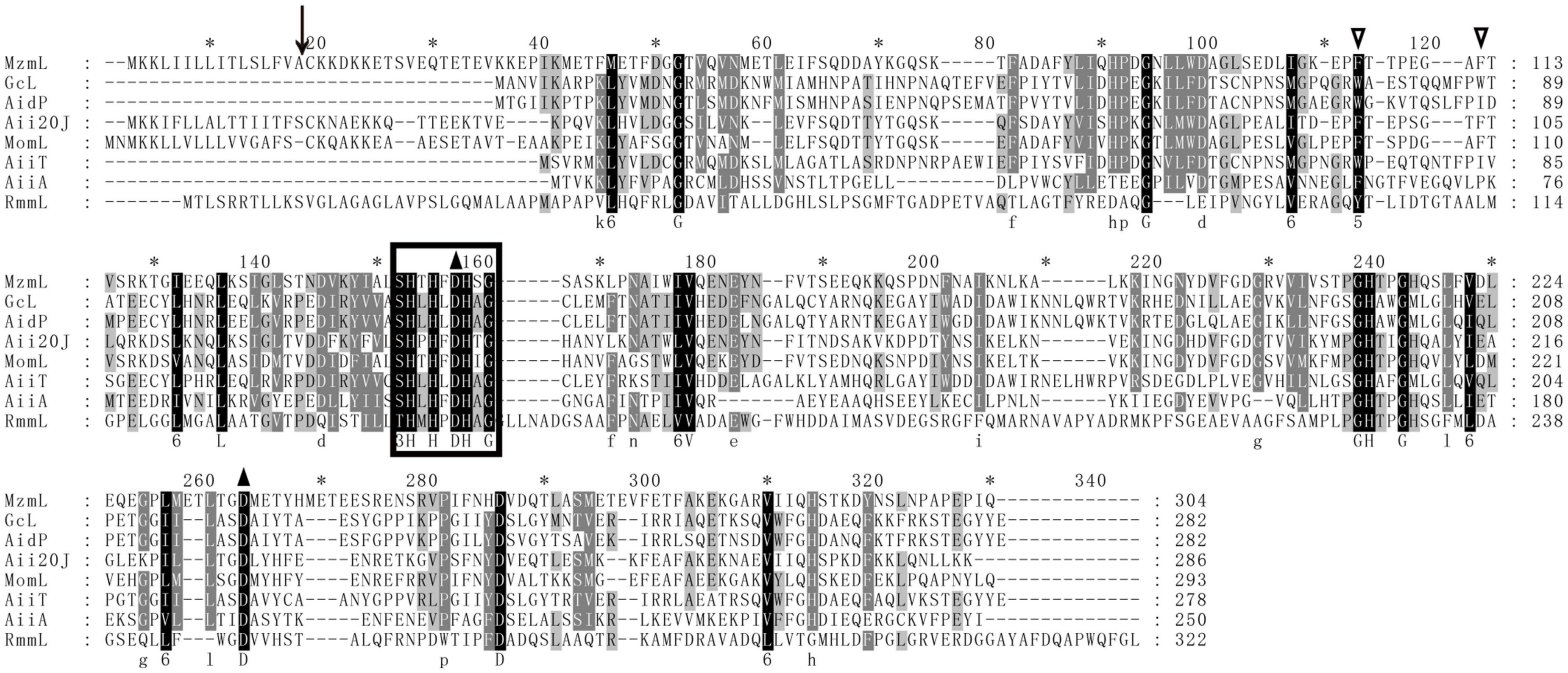
Multiple-sequence alignment of amino acid sequences of MzmL, putative homologues, and other representative AHL lactonases (GcL, AidP, Aii20J, MomL, AiiT, AiiA, RmmL). Sequence alignment was performed by the ClustalW program in the MEGA software and sequences were shaded with the Genedoc program. The identical and similar residues are shaded in black and grey, respectively. Predicted cutting site for putative signal peptide is shown by a black arrow. Empty triangles indicate the conserved two Phe residues in some lactonases. The amino acid residues that are essential for enzymatic activity of the known AHL lactonase are indicated by black triangles. The HXHXDH conserved domain found in Metallo-*β*-lactamase superfamily is circled in box.

To identify the potential lactonase encoding gene in XY-85, whole genome sequencing and local BLASTP against known QQ enzymes in the NR database was performed. One predicted ORF (GM002175), which encodes a predicted protein of 304 amino acids and was annotated as an MBL fold metallo-hydrolase (Supplementary Table S3), showed a certain degree of similarity to AiiA (identity: 34.3%), Aii20J (identity: 53.9%) and MomL (identity: 55.3%), which are previously reported AHL lactonases from *Bacillus* sp. 240B1, *Tenacibaculum* sp. 20J and *Muricauda olearia*, respectively (Valente et al., 2017). In addition, a “HXHXDH” zinc-binding motif, which is a conserved sequence in the Metallo-*β*-lactamase superfamily, was also found in the amino acid sequence of GM002175 (Figure 2). Two Phe residues (F105 and F112, indicated by empty triangles) that are related to AHL substrate specificity, as well as those that are known to be important for lactonase catalyzation, such as D145 and D237 (indicated by black triangles), were also found in the amino acid sequence of GM002175 (Tang et al., 2015). Based on these, we speculated that GM002175 could encode a novel marine-derived AHL lactonase, and named it as MzmL (*Mesoflavibacter zeaxanthinifaciens* marine lactonase).

**Figure 3:**
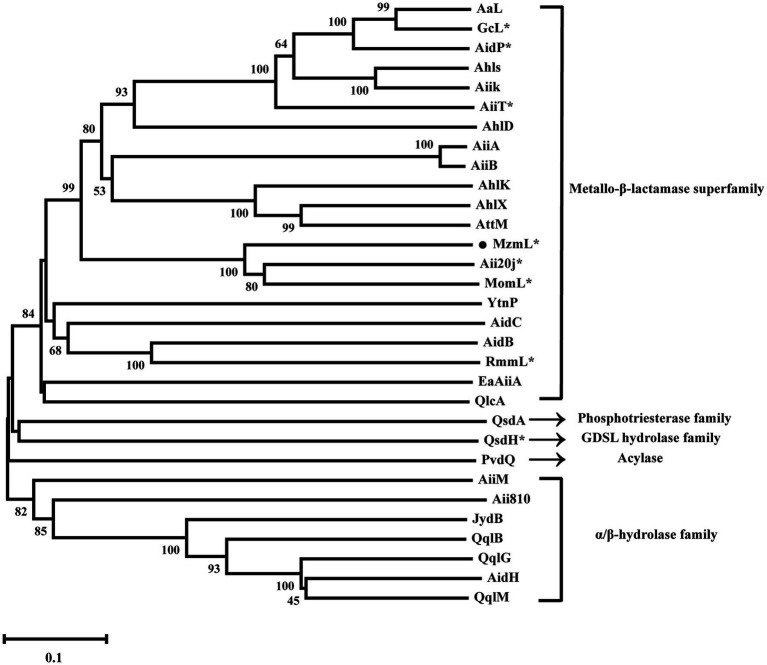
Phylogenetic analysis of MzmL and previously characterized QQ enzymes (AaL from *Alicyclobacter acidoterrestris*, GcL from *Parageobacillus caldoxylosilyticus*, AidP from *Antarctic Planococcus* sp., AhlS from *Solibacillus silvestris StLB046*, AiiK from *Kurthia huakuii*, AiiT from *Thermaerobacter nagasakiensis*, AhlD from *Arthrobacter* sp. *IBN110*, AiiA from *Bacillus* sp. *240B1*, AiiB from *Agrobacterium tumefaciens*, AhlK from *K. pneumoniae KCTC2241*, AhlX from *Salinicola salaria MCCC1A01339*, AttM from *Agrobacterium tumefaciens*, Aii20J from *Tenacibaculum* sp. *20J*, MomL from *Muricauda olearia*, YtnP from *Bacillus paralicheniformis*, AidC from *Chryseobacterium* sp. *StRB126*, AidB from *Bosea thiooxidans*, RmmL from *Ruegeria mobilis YJ3*, EaAiiA from *Erwinia amylovora*, QlcA from Soil metagenome, QsdA from *Rhodococcus erythropolis*, QsdH from *Pseudoalteromonas byunsanensis*, PvdQ from *Pseudomonas aeruginosa*, AiiM from *Microbacterium testaceum*, Aii810 from Mao-tofu metagenome, JydB from *Rhodococcus* sp. *BH4*, QqlB from *Paraburkholderia glathei*, QqlG from *Geminicoccus roseus*, AidH from *Ochrobactrum* sp. *T63*, QqlM from *Mesorhizobium cicero*). Phylogenetic analysis was conducted with the MEGA-X program with neighbor-joining method (1,000 bootstrap replicates) to show the phylogenetic relationship between MzmL and the previously reported QQ enzymes. The ^*^indicates marine derived enzymes. Bootstrap coefficients below 50% were not presented. Scale bar, 0.1 substitutions per amino acid position.

Previous studies have classified lactonases into four clusters, including the Metallo-β-lactamase superfamily, the phosphotriesterase (PTE) family, the GDSL hydrolase family, and the α/β hydrolase family (Cai et al., 2018). Phylogenetic analysis of MzmL revealed that it was closely related to the marine lactonase cluster members, Aii20J and MomL, but distant from the other lactonases such as AiiA or AidC (Figure 3). In addition, MzmL was predicted to be extracellular with an *N*-terminal signal peptide of 16 amino acid residues (MKKLIILLITLSLFVA) using SignalP 5.0 analysis, and the cleavage site was predicted between pos. 16 and 17.

### Expression, purification and bioactivity confirmation of MzmL

The recombinant MzmL was then cloned into PET-28a and expressed in *E. coli* BL21 (DE3). The purified His-tag fusion protein His-MzmL produced a single band of ~35 kDa in size, consistent with the predicted molecular weight (Supplementary Figure S3).

**Figure 4:**
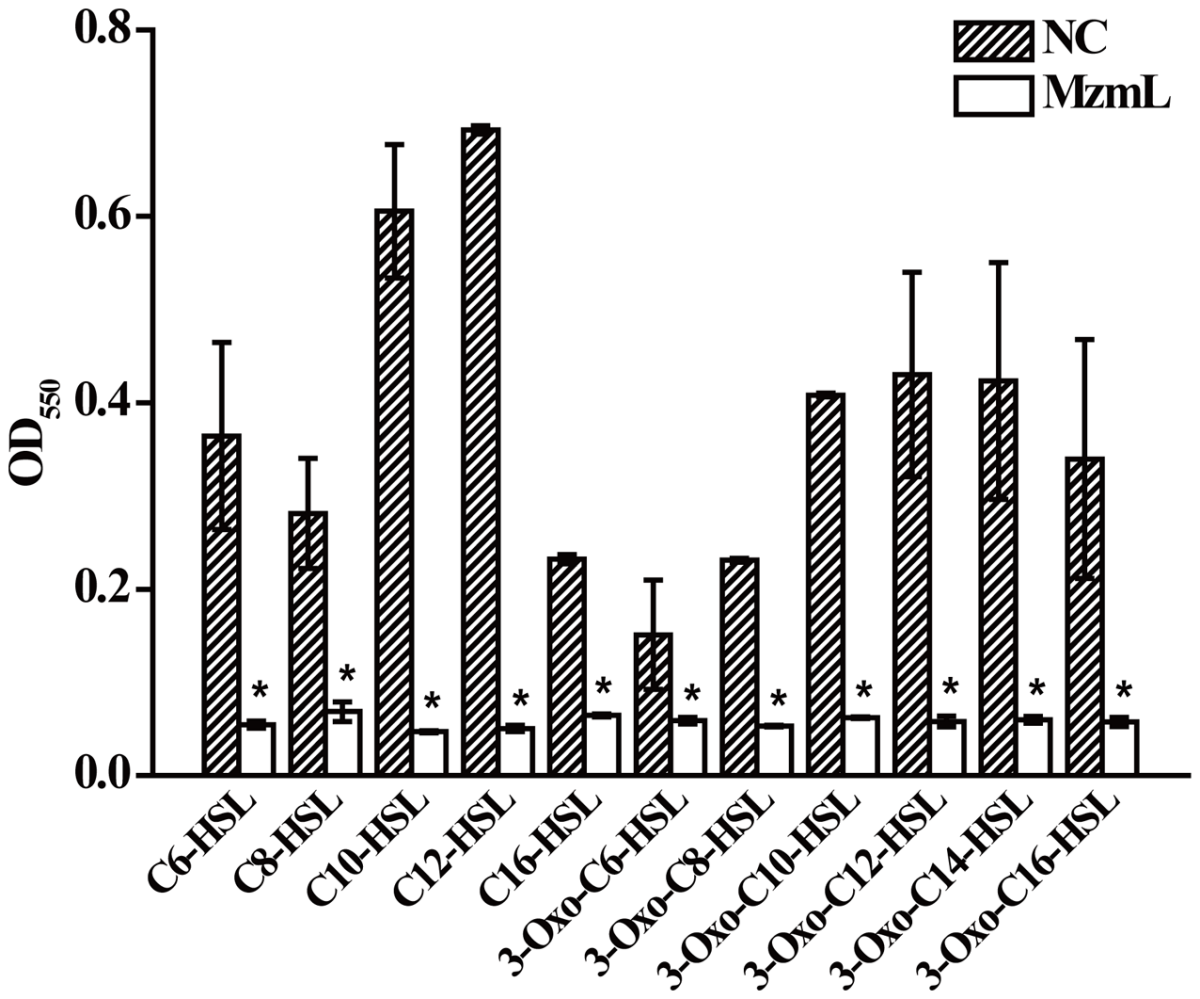
Degradative activity of MzmL towards different AHLs. The final concentration of C_6_-HSL, 3-oxo-C_6_-HSL, C_8_-HSL, 3-oxo-C_8_-HSL, C_10_-HSL, 3-oxo-C_10_-HSL, C_12_-HSL, 3-oxo-C_12_-HSL, 3-oxo-C_14_-HSL, C_16_-HSL and 3-oxo-C_16_-HSL was 20 μΜ. The final concentration of MzmL was 3 μM. The mean values were calculated based on two replicates from two independent experiments and the bars were standard errors. ^*^Indicates a significant difference of *p* < 0.01 by student’s *t*-test.

The purified MzmL can degrade both short- and long-chain AHLs (Figure 4), confirming its AHL degradative bioactivity. In addition, the kinetic activity results showed that MzmL seemed to have better efficiency towards long chain AHLs such as C_10_-HSL than C_6_-HSL (Table 2).

**Table 2:** Kinetic constants for hydrolysis of different AHLs by MzmL.

Substrate	K_m_ (mM)/Mean ± SD
C_6_-HSL	0.39 ± 0.11
3-oxo-C_6_-HSL	0.08 ± 0.06
3-oxo-C_8_-HSL	0.46 ± 0.21
C_10_-HSL	0.04 ± 0.01
3-oxo-C_10_-HSL	0.12 ± 0.03

Reactions were carried out at pH 7.1 and 28°C. The mean values were based on at least eight replicates from two independent experiments.

### UPLC-MS analysis revealed that MzmL is an *N*-acyl homoserine lactonase

**Figure 5:**
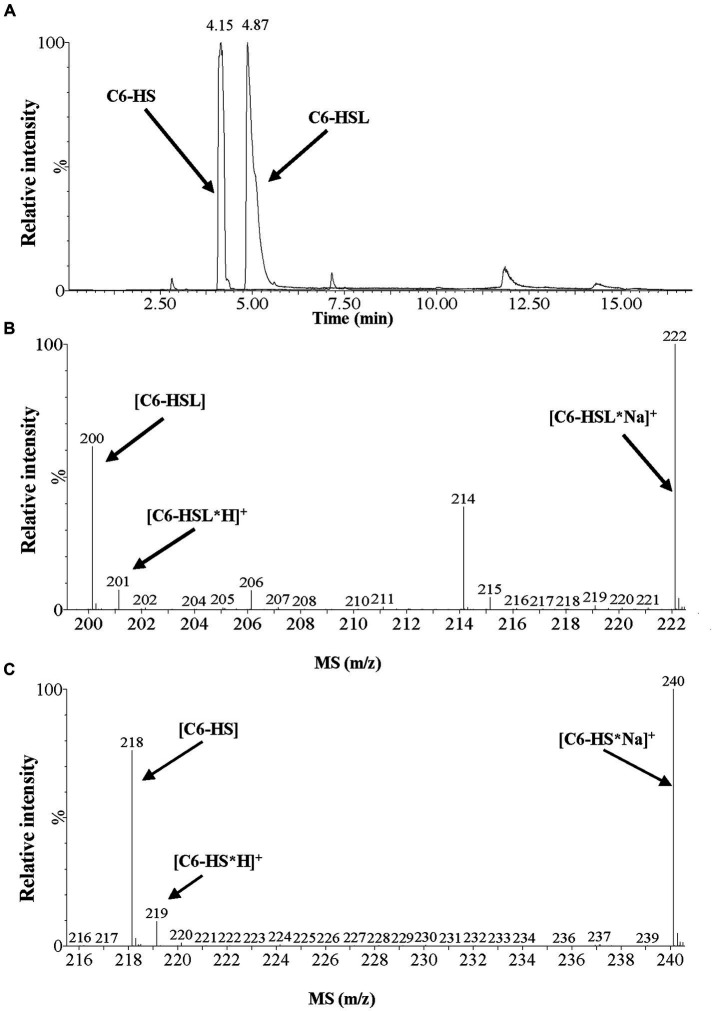
UPLC-MS analysis of the MzmL-hydrolyzed C_6_-HSL product. **(A)** UPLC profile of the MzmL-hydrolyzed C_6_-HSL product. Retention time 4.15 min, C_6_-HSL hydrolyzed 24 h by MzmL; retention time 4.87 min, undigested C_6_-HSL. **(B,C)** ESI-MS analysis of UPLC fractions containing **(B)** undigested C_6_-HSL and **(C)** C_6_-HSL hydrolyzed 24 h by MzmL.

To confirm whether MzmL is a lactonase, C_6_-HSL were digested by MzmL, and the reaction products were analyzed by UPLC-MS. The enzymatic digestion of C_6_-HSL resulted in one product with a retention time of 4.15 min, as determined by the UPLC analysis (Figure 5A). ESI-MS analysis of the product revealed a strong quasimolecule (M-H) ion at an m/z (mass-to-charge ratio) of 218, suggesting that the enzymatic action with C_6_-HSL (M-H ion m/z of 200) resulted in a mass increase of 18, corresponding to a water molecule (Figures 5B,C). This is consistent with the M-H ion m/z of the lactone-opened C_6_-HSL, namely, N-hexanoyl-l-homoserine (C_6_-HS, M-H ion m/z of 218). ESI-MS analysis revealed that the composition of the UPLC peak with a retention time of 4.87 min was undigested C_6_-HSL. These results strongly suggest that MzmL is an AHL lactonase that hydrolyzes the ester bond of the homoserine lactone ring of AHL.

### Effects of temperature, pH and metal ions on the residual enzymatic activity of MzmL

**Figure 6:**
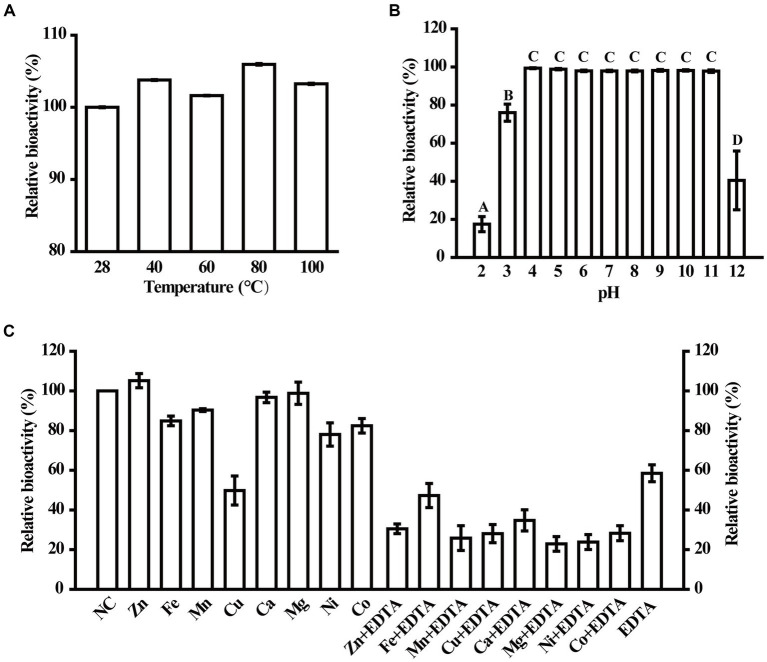
Physical and chemical parameters that affect MzmL activity. The temperature **(A)** and pH stability **(B)** of MzmL, and the effects of metal ions on MzmL activity **(C)** were determined. The mean values were calculated based on at least two replicates from two independent experiments and the bars were standard errors. Different letters represent a significant difference of *p* < 0.05 by Tukey’s honest significant difference test.

To determine the environmental stability of MzmL, the effects of several physical and chemical parameters including temperature, pH, EDTA and metal ions on its AHL degrading bioactivity were investigated. As shown in Figure 6A, MzmL was heat stable between 28–100°C, and retained 100% activity even after being heated for 30 min at 100°C. The enzyme also demonstrated good tolerance to a wide range of pH (Figure 6B), that its relative bioactivity was unaffected by pH of 4–11, and was slightly reduced to ~75% under pH of 3. When the pH was adjusted to 12 and 2, the relative activity of MzmL was reduced to ~40 and ~20%, respectively.

At 100 μM, Cu^2+^ significantly reduced the activity of MzmL, whereas Co^2+^, Ni^2+^, Mn^2+^ and Fe^2+^ only had minor effects. In contrast, Mg^2+^, Ca^2+^, and Zn^2+^ had no effects on MzmL activity (Figure 6C). When treated with 1 mM EDTA, approximately ~40% of the activity of MzmL was lost, which could not be recovered by reincubation with any of the metal ions listed above. These results indicated that MzmL has good environmental stability and can tolerate a wide range of temperature and pH, and may have a different AHL degrading mechanism from other lactonases.

### MzmL decreased virulence factor production of *Pcc in vitro* and attenuated bacterial virulence in potato slices

**Figure 7:**
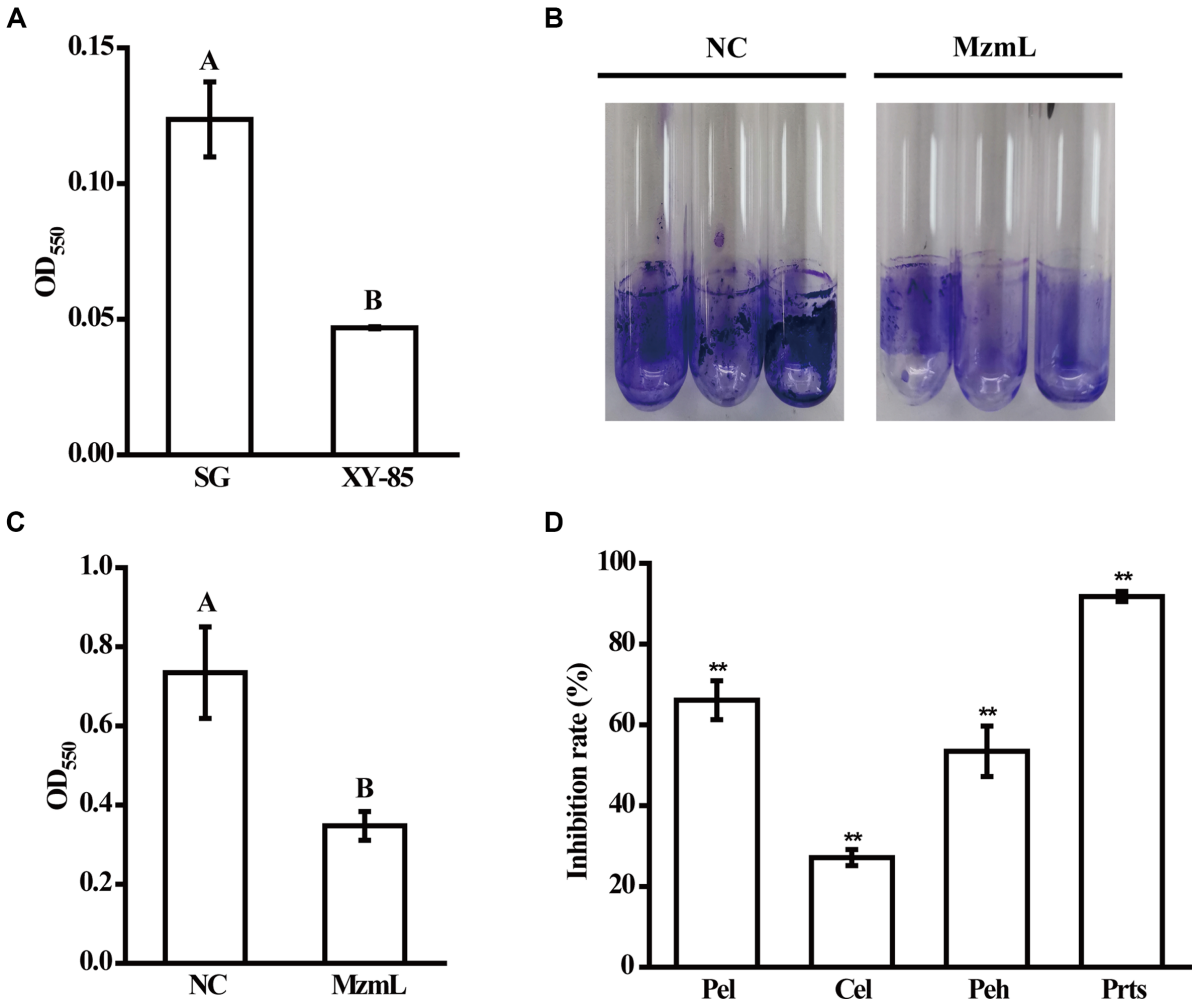
The effects of MzmL on AHLs **(A)**, biofilm **(B,C)** and plant cell wall degrading enzyme production **(D)** of *Pcc*. Pel, pectate lyase; Peh, polygalacturonase; Cel, cellulase; Prts, extracellular protease. Four replicates were included for each treatment and the experiment was repeated three times. Different letters represent a significant difference of *p* < 0.05 by Tukey’s honest significant difference test. ^**^Represents a significant difference of *p* < 0.01 comparing to 0% inhibition rate by student’s *t*-test.

**Figure 8:**
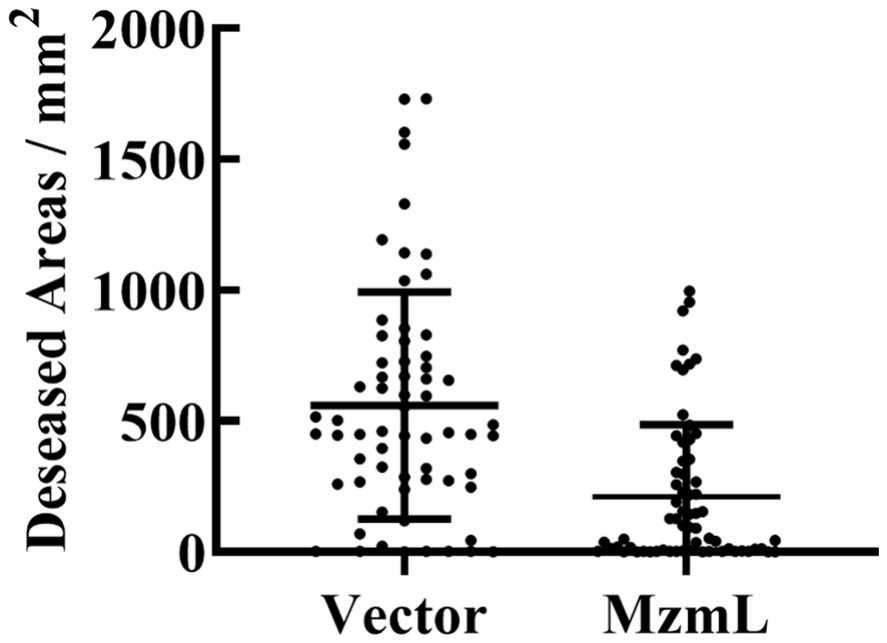
Diseased areas in potato slices co-inoculated with *Pcc* and *E. coli* BL21 (DE3) pET28a (vector), and *E. coli* BL21 (DE3) pET28a*::N6His:mzmLlac* (MzmL) respectively. Mean values were calculated based on a total of 63 replicates from three different experiments. Different letters represent a significant difference of *p* < 0.01 by student’s *t*-test.

To investigate whether MzmL had any potential in inhibition of pathogen virulence, the effects of the lactonase on virulence factor production in *Pcc* were evaluated *in vitro*. XY-85 culture was able to degrade the extracted AHL signals of *Pcc* after 24 h of incubation (Figure 7A). At 5 ppm, MzmL significantly reduced biofilm production of *Pcc* by ~52% (Figures 7B,C). When MzmL was co-cultured with *Pcc* for 24 h, several extracellular pathogenic enzyme production of the pathogen was significantly reduced. The inhibition rates on pectate lyase (Pel), polygalacturonase (Peh), cellulase (Cel) and extracellular protease (Prts) production were ~66.1, 53.5, 27.1, 91.8%, respectively (Figure 7D). When potato slices was co-inoculated with *Pcc* and *E. coli* BL21 (DE3) pET28a*::N6His:mzmLlac*, the watery rotten areas caused by the pathogen were significantly reduced compared to those co-inoculated with *Pcc* and *E. coli BL21* (DE3) pET28a (Figure 8, *p* < 0.01), while the pathogen population was not significantly different between the two treatments (Supplementary Figure S4, *p* > 0.05). These results suggested that MzmL was capable of inhibiting *Pcc* virulence factor production and virulence in plant tissues through degrading its quorum sensing signals.

## Discussion

In the past decades, bacteria with QQ bioactivities have been shown to be widely distributed in the ocean. For example, Romero et al. have reported that approximately 14.4% bacterial isolates from marine biofilm, brown seaweed and sedimental samples collected from a natural coastal environment in Illa de Arousa were capable of degrading C_6_-HSL, and ~18% isolates from the shallow seawater by the coastal line and from the Atlantic Ocean were capable of degrading C_12_-HSL (Romero et al., 2011). Our results were consistent with these findings, that ~16% bacterial strains isolated from a *Onchidium* sp., which usually dwells in shallow water zone, have C_6_-HSL degrading bioactivities. Bacterial identification results assigned the 32 isolates with QQ bioactivities obtained from initial screening to 14 genera. Five of them was shown to be QQ bioactive for the first time, including *Neptunomonas*, *Pararhodobacter*, *Pseudooceanicola*, *Pseudoreugeria*, *Sphingorhabdus*. The identification results also revealed that the symbiotic bacteria associated with this type of marine invertebrate is highly diversified, that a total of 53 genera has been identified from less than 200 isolates. Taken together, these findings suggested that marine invertebrates such as *Onchidium* sp. host superior microbial diversity and could be promising sources for mining novel QQ bioactive compounds.

The results from the bioreporter assay, initial HPLC analysis and acidification assay together suggested that the QQ compound produced by XY-85 might be a lactonase, which was subsequently confirmed by identification and characterization of a lactonase encoding gene, *mzmL*. Although the amino acid sequence of MzmL did not show high similarities to the best known AiiA type of lactonases (<35% compared to AiiA), it showed a relatively high level of sequence identity to Aii20J and MomL (~53–55%), two marine lactonases identified from marine sediment and flounder mucus, respectively (Mayer et al., 2015; Tang et al., 2015). Environmental stability results of these proteins suggested that MzmL is more similar to Aii20J but quite different from MomL. For example, MzmL was thermostable after being exposed to the 100°C for 30 min. This is comparable to the native, truncated Aii20J, which also retained full bioactivity after a treatment of 100°C for 10 min. Furthermore, the bioactivity of MzmL was unaffected by a wide range of pH from 4 to 11, and AiiJ was reported to be tolerant to a similar but relatively narrower range of pH (3–9). In contrast, MomL lost more than 70% bioactivity under 60°C or higher temperatures, and could only tolerate a pH range from 7–11. Two features in amino acid composition have been reported in thermophilic enzymes. One is increased number of proline or reduced number of glycine residues, which has been proposed as one of the factors contributing to the thermostability of phosphotriesterase-like lactonases (Hawwa et al., 2009). Another is increased number of arginine and aspartate residues in the nonconserved domains, which was seen in AiiT, another marine derived thermostable lactonase from *Thermaerobacter marianensis* that was isolated from the Mariana Trench (Morohoshi et al., 2015). Neither of these characteristics was found in MzmL, and these were also absent from Aii20J. Therefore, the thermostability of MzmL should be attributed to different features, and further site directed mutagenesis will help to clarify the key residues responsible for this character. Lastly, several other amino acid residues that are functionally crucial were also identified in MzmL. For example, tyrosine (Y194) and aspartic acid (D108) in AiiA, which are directly involved in the catalytic mechanism, were also present in MzmL, corresponding to Y241 and D145, respectively (Charendoff et al., 2013; Tang et al., 2015). In addition, Asp237 (D237) of MzmL, which was homologous to D191 of AiiA, D232 in MomL, D227 in Aii20J, is important in the formation of zinc bridging.

UPLC-MS analysis demonstrated that the mass spectrum of the degraded product of C_6_-HSL treated with MzmL produced a correct peak corresponding to acyl homoserine at 218 m/z. This confirmed that MzmL is an AHL lactonase. MzmL could degrade both short- and long-chain AHLs, but was somewhat more effective at degrading long chain signals as revealed by the kinetic assay results (Table 2). This is similar to MomL and Aii20J (Mayer et al., 2015; Tang et al., 2015). However, the metal-binding capability of MzmL is different from known lactonases, such as RmmL, AidC and MomL, in that the inhibitory effects on its bioactivity by EDTA could not be restored by addition of any of the tested ions. Whether MzmL has a different AHL-degrading mechanism from other lactonase remains to be elucidated. Further studies such as metal contents and kinetic constants of MzmL and site directed mutagenesis will help to clarify the mechanism.

The bacterial pathogen *Pcc* causes soft rot disease mainly depending on the production of a number of plant cell degrading enzymes, such as pectate lyase, pectin lyase, polygalacturonase, cellulase, and protease. These enzymes together macerate plant tissues, leading to symptom development. Biofilm formation is also important for *Pectobacterium* sp. virulence, which is critical for the pathogens to occlude xylem tissues (Moleleki et al., 2017). Both the production of exoenzymes and biofilm formation by *Pcc* is regulated with the QS system through a main signaling molecule, 3-oxo-C_6_-HSL (Fan et al., 2020), which can be degraded by MzmL. As expected, MzmL significantly reduced biofilm formation and exoenzyme production of *Pcc*. In addition, *E. coli* transformants expressing MzmL significantly inhibited soft rot development on potato slices, suggesting its potential in plant bacterial disease management. Further investigation on how to ensure stable expression and production of this enzyme *in planta*, such as using plant growth promoting bacteria as hosts, will facilitate the development of applicable biological control agents.

To dates, more than 50 AHL lactonases have been reported and identified, but only 7 were from marine resources. In this study, a novel QQ enzyme of AHL lactonase, MzmL, from *Mesoflavibacter zeaxanthinifaciens* XY-85 strain was identified and characterized. To the best of our knowledge, this is the first report of a QQ enzyme identified and purified from this genus. The good physical chemical properties of MzmL, such as tolerance to a wide range of temperatures and pH, as well as good enzymatic efficiency, suggested it has great potential to be applied in the future.

## Conclusion and future prospective

In this study, we reported bacterial isolates of *Neptunomonas*, *Pararhodobacter*, *Pseudooceanicola*, *Pseudoreugeria*, and *Sphingorhabdus* sp. having QQ bioactivity for the first time. We further identified and characterized a lactonase, MzmL, from an isolate, *Mesoflavibacter zeaxanthinifaciens* XY-85. MzmL could degrade different AHLs and retained full bioactivity under a wide range of temperatures (28–100°C) and pHs (4–11). The enzyme could also significantly reduce *Pectobacterium caratovorum* subsp. *caratovorum* virulence factor production *in vitro*, and inhibit soft rot development in plant tissues. This study provides useful information on the potential of a novel marine derived QQ enzyme in plant bacterial disease management.

## Data availability statement

The datasets presented in this study can be found in online repositories. The names of the repository/repositories and accession number(s) can be found in the article/Supplementary material.

## Author contributions

LH: Conceptualization, Formal analysis, Funding acquisition, Investigation, Methodology, Project administration, Supervision, Writing – original draft, Writing – review & editing. JL: Conceptualization, Investigation, Methodology, Validation, Writing – review & editing, Formal analysis. SC: Formal analysis, Investigation, Writing – review & editing, Validation. JZ: Formal analysis, Investigation, Writing – review & editing. YZ: Data curation, Formal analysis, Funding acquisition, Writing – review & editing. YX: Conceptualization, Funding acquisition, Project administration, Supervision, Writing – review & editing.

## Footnotes

^1^http://www.ncbi.nlm.nih.gov/BLAST/

## Supplementary material

SUPPLEMENTARY FIGURE S1 Detection of the quorum quenching bioactivity of 32 bacterial isolates associated with an *Onchidium* sp. Shown are **(A)** mean violacein production and **(B)** endpoint optical density readings of *Chromobacterium violaceum* CV026 after overnight incubation with the reaction supernatants from C_6_-HSL pre-incubated with different bacterial culture. The mean values were calculated based on twelve replicates from three independent experiments and the bars were standard errors.

SUPPLEMENTARY FIGURE S2 High-performance liquid chromatography analysis of C_6_-HSL incubated with XY-85 culture at 0 and 14 h, respectively. Presence of C_6_-HSL is indicated by the arrows.

SUPPLEMENTARY FIGURE S3 SDS-PAGE analysis of MzmL-6×His-tagged protein. MzmL protein bands stained by Coomassie brilliant blue are indicated by arrows. lane 1, *E. coli* BL21 (DE3) pET28a; lane 2, *E. coli* BL21 (DE3) pET28a induced by IPTG; lane 3, *E. coli* BL21 (DE3) pET28a*::N6His:mzmLlac*; lane 4, *E. coli* BL21 (DE3) pET28a*::N6His:mzmLlac* induced by IPTG; lane 5, soluble fraction of *E. coli* BL21 (DE3) pET28a*::N6His:mzmLlac* induced by IPTG; lane 6 and 7, 60 and 500 mM imidazole washed fractions after passage over NTA-Ni column, respectively. M, marker.

SUPPLEMENTARY FIGURE S4 Mean populations of *Pectobacterium caratovorum* subsp. *caratovorum* co-incubated with *E. coli* BL21 (DE3) pET28a (Vector) and *E. coli* BL21 (DE3) pET28a*::N6His:mzmLlac* (MzmL), respectively for 48 h. Mean values were calculated based on a total of nine replicates from three different experiments.
