# Supplementary figures and images for: MzmL, a novel marine derived N-acyl homoserine lactonase from Mesoflavibacter zeaxanthinifaciens that attenuates Pectobacterium carotovorum subsp. carotovorum virulence
Source: Front Microbiol. 2024 May 9;15:1353711. doi: 10.3389/fmicb.2024.1353711 (PMC11112094; doi:10.3389/fmicb.2024.1353711)

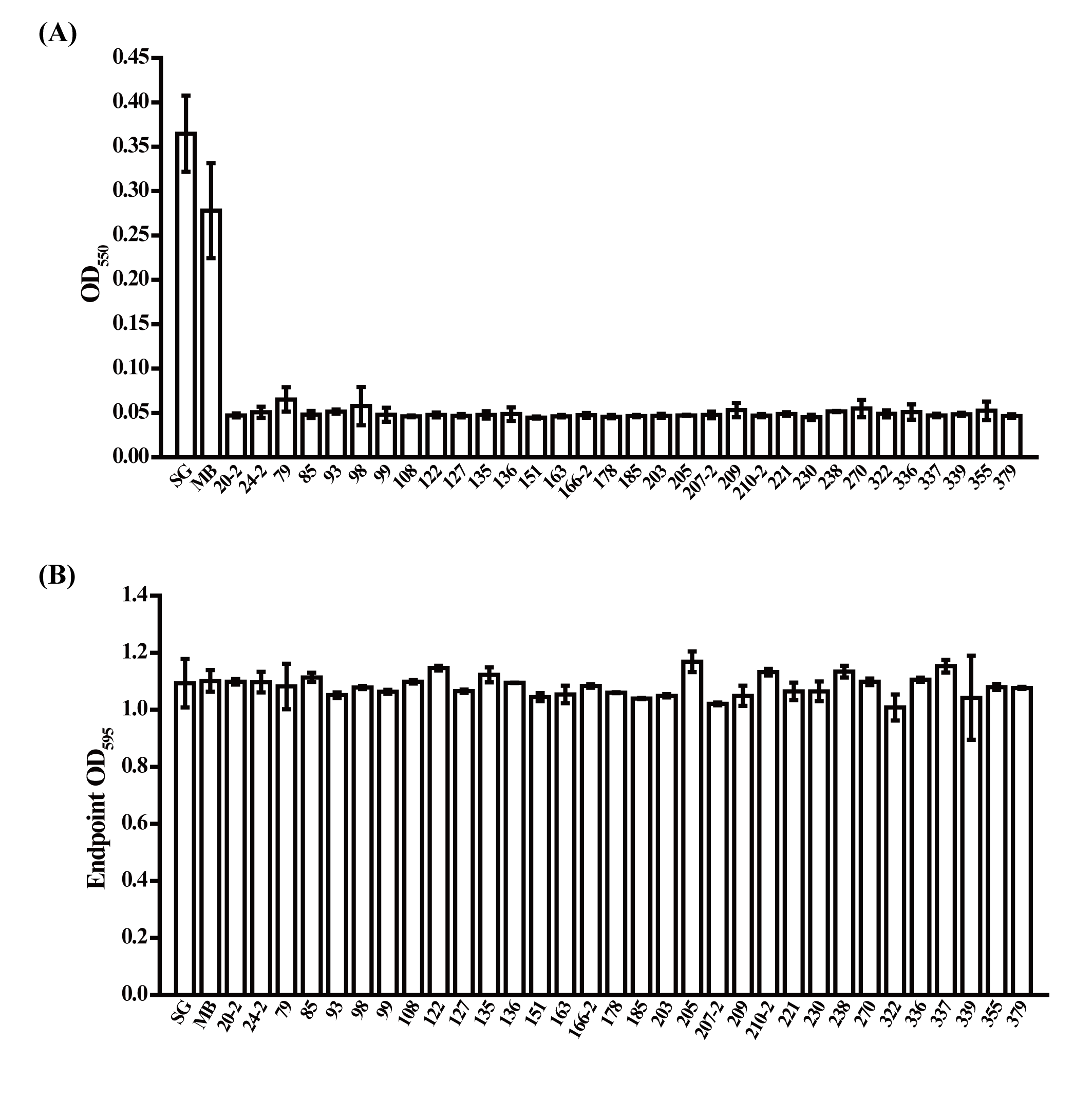

Supplement: SUPPLEMENTARY FIGURE S1 — Detection of the quorum quenching bioactivity of 32 bacterial isolates associated with an Onchidium sp. Shown are (A) mean violacein production and (B) endpoint optical density readings of Chromobacterium violaceum CV026 after overnight incubation with the reaction supernatants from C6-HSL pre-incubated with different bacterial culture. The mean values were calculated based on twelve replicates from three independent experiments and the bars were standard errors. [file Image_1.TIF]

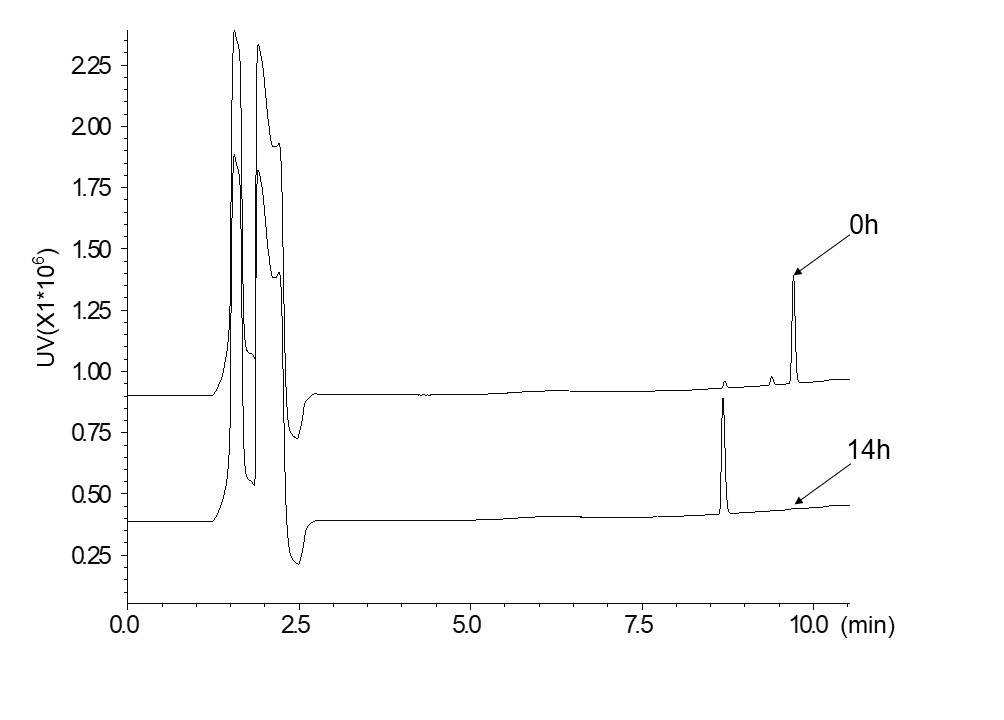

Supplement: SUPPLEMENTARY FIGURE S2 — High-performance liquid chromatography analysis of C6-HSL incubated with XY-85 culture at 0 and 14 h, respectively. Presence of C6-HSL is indicated by the arrows. [file Image_2.TIF]

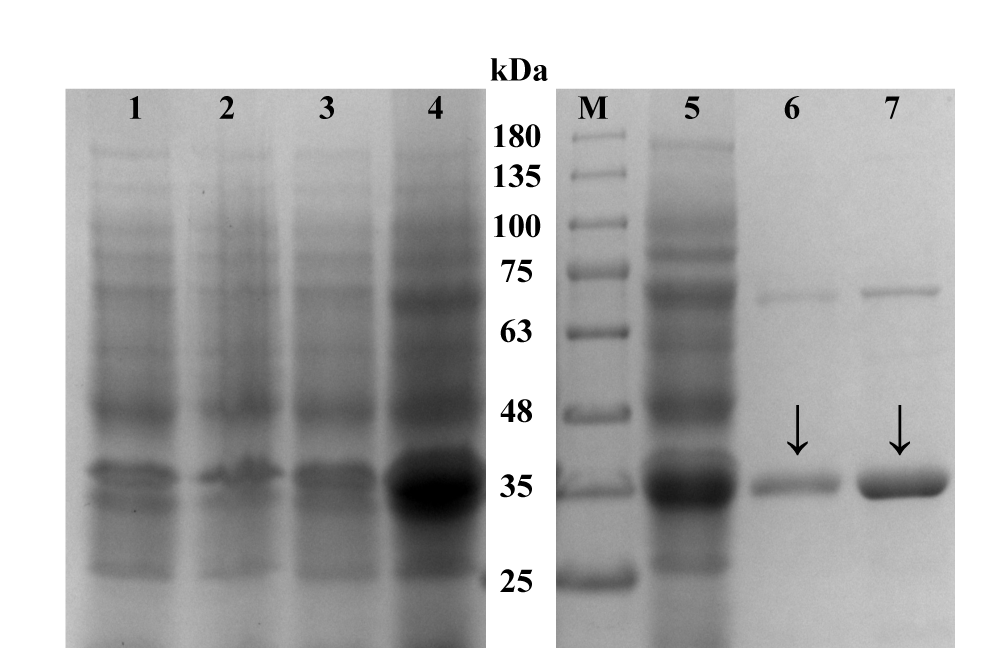

Supplement: SUPPLEMENTARY FIGURE S3 — SDS-PAGE analysis of MzmL-6×His-tagged protein. MzmL protein bands stained by Coomassie brilliant blue are indicated by arrows. lane 1, E. coli BL21 (DE3) pET28a; lane 2, E. coli BL21 (DE3) pET28a induced by IPTG; lane 3, E. coli BL21 (DE3) pET28a::N6His:mzmLlac; lane 4, E. coli BL21 (DE3) pET28a::N6His:mzmLlac induced by IPTG; lane 5, soluble fraction of E. coli BL21 (DE3) pET28a::N6His:mzmLlac induced by IPTG; lane 6 and 7, 60 and 500 mM imidazole washed fractions after passage over NTA-Ni column, respectively. M, marker. [file Image_3.TIF]

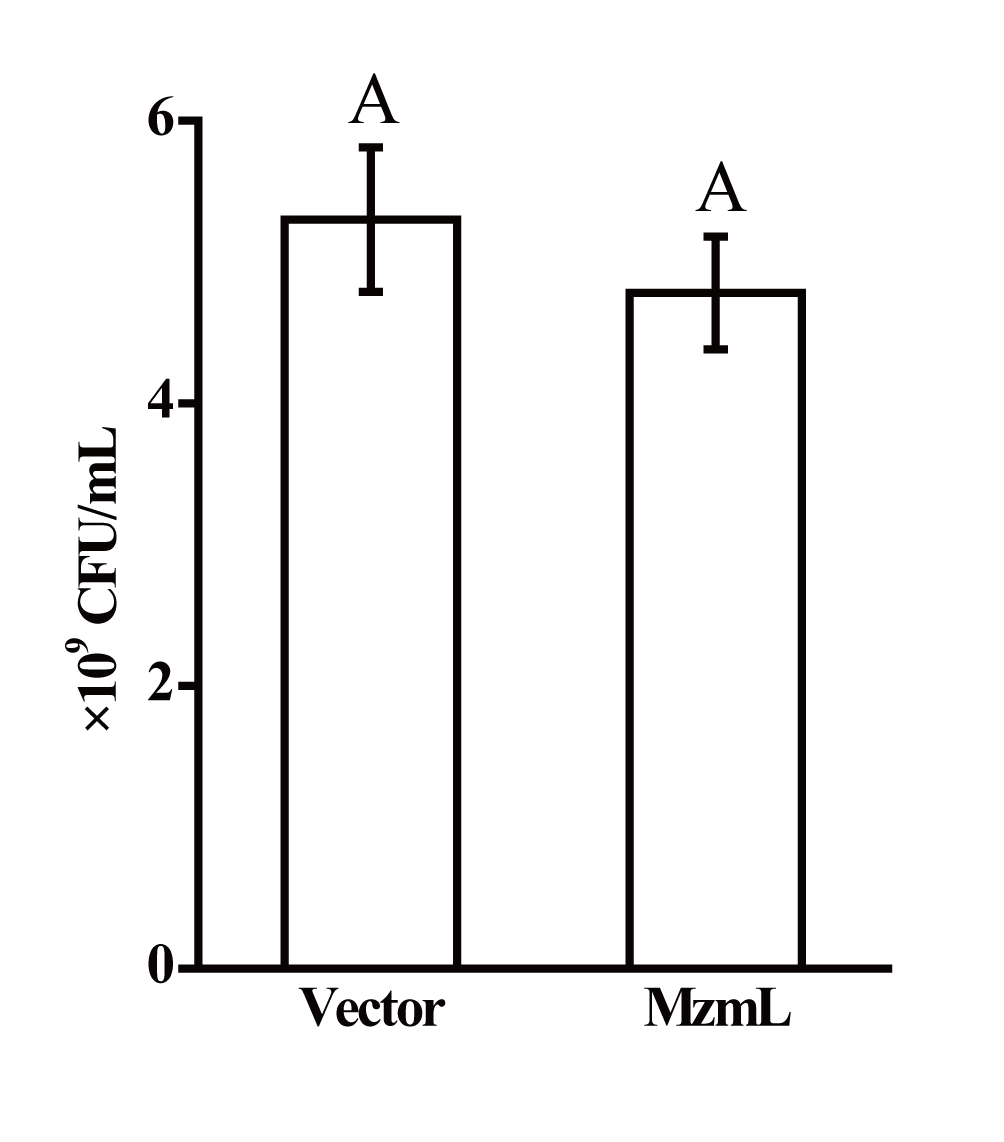

Supplement: SUPPLEMENTARY FIGURE S4 — Mean populations of Pectobacterium caratovorum subsp. caratovorum co-incubated with E. coli BL21 (DE3) pET28a (Vector) and E. coli BL21 (DE3) pET28a::N6His:mzmLlac (MzmL), respectively for 48 h. Mean values were calculated based on a total of nine replicates from three different experiments. [file Image_4.TIF]
