# Supplementary material for: MzmL, a novel marine derived N-acyl homoserine lactonase from Mesoflavibacter zeaxanthinifaciens that attenuates Pectobacterium carotovorum subsp. carotovorum virulence
Source: Front Microbiol. 2024 May 9;15:1353711. doi: 10.3389/fmicb.2024.1353711 (PMC11112094; doi:10.3389/fmicb.2024.1353711)
Supplement: Supplementary file 5 [file Table_1.DOCX]

Table 1. Summary of classified bacterial isolates with or without quorum quenching (QQ) bioactivities associated with an *Onchidium* sp. collected from Dapeng Bay at Shenzhen, China.

|  | Genus | No. of isolates | Strain number | QQ bioactivity |
| --- | --- | --- | --- | --- |
|  | ***Acinetobacter*** | 1 | XY-205 | **√** |
|  |  | 1 | XY-91 | × |
|  | *Ahrensia* | 2 | XY-113, 222 | × |
|  | *Algibacter* | 1 | XY-114 | × |
|  | *Alteromonas* | 3 | XY-153, 165, 170 | × |
|  | *Alteromonas* | 4 | XY-150, 169, 174, 187 | × |
|  | *Antarctobacter* | 1 | XY-81 | × |
|  | *Aquimarina* | 1 | XY-208 | × |
|  | ***Bacillus*** | 6 | XY-20-2, 24-2, 79, 93, 98, 379 | √ |
|  |  | 27 | XY-25, 32, 35, 37, 40, 41, 43, 46, 48, 51, 55, 58, 63, 65, 68, 69, 95, 102, 106, 111, 128, 186, 191, 199, 224, 228, 362 | × |
|  | *Brevibacterium* | 2 | XY-216, 256 | × |
|  | *Brevundimonas* | 3 | XY-252, 364, 369 | × |
|  | *Cellulophaga* | 3 | XY-154, 157, 158 | × |
|  | *Cellvibrio* | 1 | XY-145 | × |
|  | *Donghicola* | 1 | XY-67 | × |
|  | *Echinicola* | 1 | XY-200 | × |
|  | ***Erythrobacter*** | 5 | XY-185, 207-2, 230, 238, 336 | √ |
|  | *Fangia* | 1 | XY-144 | × |
|  | *Flavobacterium* | 1 | XY-259 | × |
|  | *Glutamicibacter* | 2 | XY-162, 184 | × |
|  | *Labrenzia* | 2 | XY-192, 355-2 | × |
|  | *Lacinutrix* | 4 | XY-73, 83, 112, 116 | × |
|  | *Leisingera* | 1 | XY-116 | × |
|  | *Leucobacter* | 2 | XY-143-2, 382 | × |
|  | *Loktanella* | 1 | XY-334-1 | × |
|  | *Luteimonas* | 3 | XY-21, 131, 345 | × |
|  | *Lysobacter* | 1 | XY-343 | × |
|  | *Maribacter* | 3 | XY-87, 110, 176, 325 | × |
|  | *Marinomonas* | 1 | XY-23 | × |
|  | ***Mesoflavibacter*** | 7 | XY-85, 108, 122, 135, 136, 166-2, 221 | √ |
|  | ***Microbacterium*** | 3 | XY-151, 203, 209 | √ |
|  | *Microbulbifer* | 1 | XY-92 | × |
|  | ***Neptunomonas*** | 1 | XY-337 | √ |
|  | *Nonlabens* | 4 | XY-118, 172-1, 175, 179 | × |
|  | *Ochrobactrum* | 1 | XY-130 | × |
|  | ***Paracoccus*** | 1 | XY-322 | √ |
|  |  | 10 | XY-20-1,30, 34, 146, 148, 167, 172-2, 180, 182, 330 | × |
|  | ***Pararhodobacter*** | 1 | XY-270 | √ |
|  | *Phaeobacter* | 1 | XY-126-1 | × |
|  | *Pseudoalteromonas* | 1 | XY-133 | × |
|  | ***Pseudooceanicola*** | 1 | XY-99 | √ |
|  | ***Pseudoruegeria*** | 1 | XY-210-2 | √ |
|  |  | 1 | XY-164-2 | × |
|  | *Pseudovibrio* | 9 | XY-45-1, 49, 50, 53, 59, 90, 96, 104, 107 | × |
|  | ***Rhodococcus*** | 1 | XY-127 | √ |
|  | ***Roseomonas*** | 1 | XY-339 | √ |
|  | *Roseovarius* | 1 | XY-28 | × |
|  | *Ruegeria* | 20 | XY-27, 84, 86, 101, 109, 137, 142, 152, 159, 181-2, 189, 220-1, 247, 300, 319, 321, 323, 328, 361, 363 | × |
|  | *Sagittula* | 1 | XY-166-1 | × |
|  | *Salinimonas* | 1 | XY-15-2 | × |
|  | *Shimia* | 2 | XY-123, 333 | × |
|  | ***Sphingorhabdus*** | 1 | XY-355 | √ |
|  | *Sulfitobacter* | 21 | XY-66, 71, 72, 78, 89, 115, 119, 125-2, 164-1, 173-1, 177, 181-1, 188, 190, 197, 202, 204, 206, 207, 269, 331 | × |
|  | ***Tenacibaculum*** | 2 | XY-163, 178 | √ |
|  |  | 6 | XY-29, 56, 117, 156, 160, 171 | × |
|  | *Thalassococcus* | 1 | XY-70-1 | × |
|  | *Tropicibacter* | 1 | XY-155 | × |
|  | *Vibrio* | 8 | XY-26, 39, 47, 54, 94, 134, 140, 367 | × |
|  | *Zobellia* | 2 | XY-121, 193 | × |
| Total No. | 54 | 197 |  | 32 |

Table 1. Shown is the classification and counting of all bacterial strains with or without QQ bioactivity associated with an *Onchidium* sp. Classification was conducted based on comparison of bacterial 16s ribosomal DNA sequences with those in the NCBI database. The numbers of isolates assigned to each genus were counted. QQ bioactivity was detected by using the bioreporter strain *Chromobacterium violaceum* CV026, and the genera cotaining QQ bioactive strains are highlighted in black.
