# Supplementary material for: MzmL, a novel marine derived N-acyl homoserine lactonase from Mesoflavibacter zeaxanthinifaciens that attenuates Pectobacterium carotovorum subsp. carotovorum virulence
Source: Front Microbiol. 2024 May 9;15:1353711. doi: 10.3389/fmicb.2024.1353711 (PMC11112094; doi:10.3389/fmicb.2024.1353711)
Supplement: Supplementary file 6 [file Table_2.DOCX]

Table 2. Kinetic constants for hydrolysis of different AHLs by MzmL.

| Substrate | K_m_ (mM) / Mean ± SD |
| --- | --- |
| C_6_-HSL | 0.39 ± 0.11 |
| 3-Oxo-C_6_-HSL | 0.08 ± 0.06 |
| 3-Oxo-C_8_-HSL | 0.46 ± 0.21 |
| C_10_-HSL | 0.04 ± 0.01 |
| 3-Oxo-C_10_-HSL | 0.12 ± 0.03 |

Reactions were carried out at pH 7.1 and 28°C. The mean values were based on at least eight replicates from two independent experiments.
