# Supplementary material for: MzmL, a novel marine derived N-acyl homoserine lactonase from Mesoflavibacter zeaxanthinifaciens that attenuates Pectobacterium carotovorum subsp. carotovorum virulence
Source: Front Microbiol. 2024 May 9;15:1353711. doi: 10.3389/fmicb.2024.1353711 (PMC11112094; doi:10.3389/fmicb.2024.1353711)
Supplement: Supplementary file 7 [file Table_3.DOCX]

Table S1. List of all bacterial isolates identified from an *Onchidium* sp. sampled from Dapeng Bay in Shenzhen, China.

| Strain No. | Species with closest similarity | Accession No. |
| --- | --- | --- |
| XY-382 | *Leucobacter musarum subsp. japonicus* | OR262693 |
| XY-379 | *Bacillus wiedmannii* | OR262694 |
| XY-369 | *Brevundimonas vesicularis* | OR262695 |
| XY-367 | *Vibrio harveyi* | OR262696 |
| XY-364 | *Brevundimonas vesicularis* | OR262697 |
| XY-363 | *Ruegeria arenilitoris* | OR262698 |
| XY-362 | *Bacillus tequilensis* | OR262699 |
| XY-361 | *Ruegeria arenilitoris* | OR262700 |
| XY-355 | *Sphingorhabdus litoris* | OR262701 |
| XY-345 | *Luteimonas aestuarii* | OR262702 |
| XY-343 | *Lysobacter daecheongensis* | OR262703 |
| XY-339 | *Roseomonas mucosa* | OR262704 |
| XY-337 | *Neptunomonas concharum* | OR262705 |
| XY-336 | *Erythrobacter citreus* | OR262706 |
| XY-335-2 | *Labrenzia alexandrii* | OR262707 |
| XY-334-1 | *Loktanella rosea* | OR262708 |
| XY-333 | *Shimia marina* | OR262709 |
| XY-331 | *Sulfitobacter pontiacus* | OR262710 |
| XY-330 | *Paracoccus fistulariae* | OR262711 |
| XY-328 | *Ruegeria arenilitoris* | OR262712 |
| XY-325 | *Maribacter dokdonensis* | OR262713 |
| XY-323 | *Ruegeria arenilitoris* | OR262714 |
| XY-322 | *Paracoccus homiensis* | OR262715 |
| XY-321 | *Ruegeria conchae* | OR262716 |
| XY-319 | *Ruegeria atlantica* | OR262717 |
| XY-300 | *Ruegeria atlantica* | OR262718 |
| XY-270 | *Pararhodobacter aggregans* | OR262719 |
| XY-269 | *Sulfitobacter pontiacus* | OR262720 |
| XY-259 | *Flavobacterium jejuense* | OR262721 |
| XY-256 | *Brevibacterium sediminis* | OR262722 |
| XY-252 | *Brevundimonas vesicularis* | OR262723 |
| XY-247 | *Ruegeria scottomollicae* | OR262724 |
| XY-238 | *Erythrobacter flavus* | OR262725 |
| XY-230 | *Erythrobacter flavus* | OR262726 |
| XY-228 | *Bacillus altitudinis* | OR262727 |
| XY-224 | *Bacillus siamensis* | OR262728 |
| XY-222 | *Ahrensia marina* | OR262729 |
| XY-221 | *Mesoflavibacter zeaxanthinifaciens* | OR262730 |
| XY-220-1 | *Ruegeria mobilis* | OR262731 |
| XY-216 | *Brevibacterium sanguinis* | OR262732 |
| XY-210-2 | *Pseudoruegeria aquimaris* | OR262733 |
| XY-209 | *Microbacterium aquimaris* | OR262734 |
| XY-208 | *Aquimarina litoralis* | OR262735 |
| XY-207-2 | *Erythrobacter flavus* | OR262736 |
| XY-207 | *Sulfitobacter pontiacus* | OR262737 |
| XY-206 | *Sulfitobacter pontiacus* | OR262738 |
| XY-205 | *Acinetobacter vivianii* | OR262739 |
| XY-204 | *Sulfitobacter pontiacus* | OR262740 |
| XY-203 | *Microbacterium koreense* | OR262741 |
| XY-202 | *Sulfitobacter pontiacus* | OR262742 |
| XY-200 | *Echinicola pacifica* | OR262743 |
| XY-199 | *Bacillus altitudinis* | OR262744 |
| XY-197 | *Sulfitobacter pontiacus* | OR262745 |
| XY-193 | *Zobellia russellii* | OR262746 |
| XY-192 | *Labrenzia alexandrii* | OR262747 |
| XY-191 | *Bacillus altitudinis* | OR262748 |
| XY-190 | *Sulfitobacter pontiacus* | OR262749 |
| XY-189 | *Ruegeria scottomollicae* | OR262750 |
| XY-188 | *Sulfitobacter pontiacus* | OR262751 |
| XY-187 | *Alteromonas macleodii* | OR262752 |
| XY-186 | *Bacillus hwajinpoensis* | OR262753 |
| XY-185 | *Erythrobacter flavus* | OR262754 |
| XY-184 | *Glutamicibacter mysorens* | OR262755 |
| XY-182 | *Paracoccus zeaxanthinifaciens* | OR262756 |
| XY-181-2 | *Ruegeria mobilis* | OR262757 |
| XY-181-1 | *Sulfitobacter pontiacus* | OR262758 |
| XY-180 | *Paracoccus zeaxanthinifaciens* | OR262759 |
| XY-179 | *Nonlabens sediminis* | OR262760 |
| XY-178 | *Tenacibaculum litoreum* | OR262761 |
| XY-177 | *Sulfitobacter pontiacus* | OR262762 |
| XY-176 | *Maribacter dokdonensis* | OR262763 |
| XY-175 | *Nonlabens sediminis* | OR262764 |
| XY-174 | *Alteromonas macleodii* | OR262765 |
| XY-173-1 | *Sulfitobacter pontiacus* | OR262766 |
| XY-172-2 | *Paracoccus homiensis* | OR262767 |
| XY-172-1 | *Nonlabens tegetincola* | OR262768 |
| XY-171 | *Tenacibaculum geojense* | OR262769 |
| XY-170 | *Alteromonas australica* | OR262770 |
| XY-169 | *Alteromonas macleodii* | OR262771 |
| XY-167 | *Paracoccus homiensis* | OR262772 |
| XY-166-2 | *Mesoflavibacter sabulilitoris* | OR262773 |
| XY-166-1 | *Sagittula stellata* | OR262774 |
| XY-165 | *Alteromonas confluentis* | OR262775 |
| XY-164-2 | *Pseudoruegeria aquimaris* | OR262776 |
| XY-164-1 | *Sulfitobacter pontiacus* | OR262777 |
| XY-163 | *Tenacibaculum mesophilum* | OR262778 |
| XY-162 | *Glutamicibacter mysorens* | OR262779 |
| XY-160 | *Tenacibaculum mesophilum* | OR262780 |
| XY-159 | *Ruegeria mobilis* | OR262781 |
| XY-158 | *Cellulophaga lytica* | OR262782 |
| XY-157 | *Cellulophaga lytica* | OR262783 |
| XY-156 | *Tenacibaculum geojense* | OR262784 |
| XY-155 | *Tropicibacter naphthalenivorans* | OR262785 |
| XY-154 | *Cellulophaga geojensis* | OR262786 |
| XY-153 | *Alteromonas confluentis* | OR262787 |
| XY-152 | *Ruegeria scottomollicae* | OR262788 |
| XY-151 | *Microbacterium kitamiense* | OR262789 |
| XY-150 | *Alteromonas marina* | OR262790 |
| XY-148 | *Paracoccus zeaxanthinifaciens* | OR262791 |
| XY-146 | *Paracoccus homiensis* | OR262792 |
| XY-145 | *Cellvibrio ostraviensis* | OR262793 |
| XY-144 | *Fangia hongkongensis* | OR262794 |
| XY-143-2 | *Leucobacter musarum subsp. japonicus* | OR262795 |
| XY-142 | *Ruegeria arenilitoris* | OR262796 |
| XY-140 | *Vibrio harveyi* | OR262797 |
| XY-137 | *Ruegeria arenilitoris* | OR262798 |
| XY-136 | *Mesoflavibacter sabulilitoris* | OR262799 |
| XY-135 | *Mesoflavibacter sabulilitoris* | OR262800 |
| XY-134 | *Vibrio owensii* | OR262801 |
| XY-133 | *Pseudoalteromonas shioyasakiensis* | OR262802 |
| XY-131 | *Luteimonas aestuarii* | OR262803 |
| XY-130 | *Ochrobactrum anthropi* | OR262804 |
| XY-128 | *Bacillus altitudinis* | OR262805 |
| XY-127-1 | *Rhodococcus qingshengii* | OR262806 |
| XY-126-1 | *Phaeobacter gallaeciensis* | OR262807 |
| XY-125-2 | *Sulfitobacter pontiacus* | OR262808 |
| XY-123 | *Shimia marina* | OR262809 |
| XY-122 | *Mesoflavibacter zeaxanthinifaciens* | OR262810 |
| XY-121 | *Zobellia russellii* | OR262811 |
| XY-119 | *Sulfitobacter pontiacus* | OR262812 |
| XY-118 | *Nonlabens arenilitoris* | OR262813 |
| XY-117 | *Tenacibaculum litopenaei* | OR262814 |
| XY-116 | *Leisingera aquimarina* | OR262815 |
| XY-115 | *Sulfitobacter pontiacus* | OR262816 |
| XY-114 | *Algibacter aestuarii* | OR262817 |
| XY-113 | *Ahrensia marina* | OR262818 |
| XY-112 | *Lacinutrix venerupis* | OR262819 |
| XY-111 | *Bacillus siamensis* | OR262820 |
| XY-110 | *Maribacter dokdonensis* | OR262821 |
| XY-108 | *Mesoflavibacter sabulilitoris* | OR262822 |
| XY-109 | *Ruegeria atlantica* | OR262823 |
| XY-107 | *Pseudovibrio denitrificans* | OR262824 |
| XY-106 | *Bacillus siamensis* | OR262825 |
| XY-104 | *Pseudovibrio denitrificans* | OR262826 |
| XY-102 | *Bacillus algicola* | OR262827 |
| XY-101 | *Ruegeria arenilitoris* | OR262828 |
| XY-99 | *Pseudooceanicola nitratireducens* | OR262829 |
| XY-98 | *Bacillus anthracis* | OR262830 |
| XY-96 | *Pseudovibrio denitrificans* | OR262831 |
| XY-95 | *Bacillus algicola* | OR262832 |
| XY-94 | *Vibrio shilonii* | OR262833 |
| XY-93 | *Bacillus anthracis* | OR262834 |
| XY-92 | *Microbulbifer variabilis* | OR262835 |
| XY-91 | *Acinetobacter johnsonii* | OR262836 |
| XY-90 | *Pseudovibrio denitrificans* | OR262837 |
| XY-89 | *Sulfitobacter pontiacus* | OR262838 |
| XY-87 | *Maribacter dokdonensis* | OR262839 |
| XY-86 | *Ruegeria arenilitoris* | OR262840 |
| XY-85 | *Mesoflavibacter zeaxanthinifaciens* | OR262841 |
| XY-84 | *Ruegeria arenilitoris* | OR262842 |
| XY-83 | *Lacinutrix venerupis* | OR262843 |
| XY-81 | *Antarctobacter jejuensis* | OR262844 |
| XY-79 | *Bacillus anthracis* | OR262845 |
| XY-78 | *Sulfitobacter faviae* | OR262846 |
| XY-73 | *Lacinutrix venerupis* | OR262847 |
| XY-72 | *Sulfitobacter pontiacus* | OR262848 |
| XY-71 | *Sulfitobacter pontiacus* | OR262849 |
| XY-70-1 | *Thalassococcus lentus* | OR262850 |
| XY-69 | *Bacillus altitudinis* | OR262851 |
| XY-68 | *Bacillus altitudinis* | OR262852 |
| XY-67 | *Donghicola tyrosinivorans* | OR262853 |
| XY-66 | *Sulfitobacter pontiacus* | OR262854 |
| XY-65 | *Bacillus altitudinis* | OR262855 |
| XY-63 | *Bacillus altitudinis* | OR262856 |
| XY-59 | *Pseudovibrio denitrificans* | OR262857 |
| XY-58 | *Bacillus altitudinis* | OR262858 |
| XY-56 | *Tenacibaculum mesophilum* | OR262859 |
| XY-55 | *Bacillus altitudinis* | OR262860 |
| XY-54 | *Vibrio shilonii* | OR262861 |
| XY-53 | *Pseudovibrio denitrificans* | OR262862 |
| XY-51 | *Bacillus altitudinis* | OR262863 |
| XY-50 | *Pseudovibrio japonicus* | OR262864 |
| XY-49 | *Pseudovibrio denitrificans* | OR262865 |
| XY-48 | *Bacillus siamensis* | OR262866 |
| XY-47 | *Vibrio shilonii* | OR262867 |
| XY-46 | *Bacillus altitudinis* | OR262868 |
| XY-45-1 | *Pseudovibrio denitrificans* | OR262869 |
| XY-43 | *Bacillus siamensis* | OR262870 |
| XY-41 | *Bacillus altitudinis* | OR262871 |
| XY-40 | *Bacillus altitudinis* | OR262872 |
| XY-39 | *Vibrio variabilis* | OR262873 |
| XY-37 | *Bacillus altitudinis* | OR262874 |
| XY-35 | *Bacillus altitudinis* | OR262875 |
| XY-34 | *Paracoccus zeaxanthinifaciens* | OR262876 |
| XY-32 | *Bacillus altitudinis* | OR262877 |
| XY-30 | *Paracoccus homiensis* | OR262878 |
| XY-29 | *Tenacibaculum lutimaris* | OR262879 |
| XY-28 | *Roseovarius nubinhibens* | OR262880 |
| XY-27 | *Ruegeria arenilitoris* | OR262881 |
| XY-26 | *Vibrio harveyi* | OR262882 |
| XY-25 | *Bacillus altitudinis* | OR262883 |
| XY-24-2 | *Bacillus wiedmannii* | OR262884 |
| XY-23 | *Marinomonas communis* | OR262885 |
| XY-21 | *Luteimonas aestuarii* | OR262886 |
| XY-20-2 | *Bacillus wiedmannii* | OR262887 |
| XY-20-1 | *Paracoccus homiensis* | OR262888 |
| XY-15-2 | *Salinimonas lutimaris* | OR262889 |

Table S1. A total of 197 strains were identified and classified based on comparison of their 16s ribosomal DNA sequences with those in the NCBI database. Species with closest similarity (over 99%) and the accession number for each isolate are shown.
