# Supplementary material for: MzmL, a novel marine derived N-acyl homoserine lactonase from Mesoflavibacter zeaxanthinifaciens that attenuates Pectobacterium carotovorum subsp. carotovorum virulence
Source: Front Microbiol. 2024 May 9;15:1353711. doi: 10.3389/fmicb.2024.1353711 (PMC11112094; doi:10.3389/fmicb.2024.1353711)
Supplement: Supplementary file 8 [file Table_4.DOCX]

Table S2. Summary of all isolates from an *Onchidium* sp. showing C_6_-HSL degrading bioactivity *in vitro*.

| Strain No. | Species |
| --- | --- |
| XY-20-2 | *Bacillus wiedmannii* |
| XY-24-2 | *Bacillus wiedmannii* |
| XY-79 | *Bacillus anthracis* |
| XY-85 | *Mesoflavibacter zeaxanthinifaciens* |
| XY-93 | *Bacillus anthracis* |
| XY-98 | *Bacillus anthracis* |
| XY-99 | *Pseudooceanicola nitratireducens* |
| XY-108 | *Mesoflavibacter sabulilitoris* |
| XY-122 | *Mesoflavibacter zeaxanthinifaciens* |
| XY-127 | *Rhodococcus qingshengii* |
| XY-135 | *Mesoflavibacter sabulilitoris* |
| XY-136 | *Mesoflavibacter sabulilitoris* |
| XY-151 | *Microbacterium kitamiense* |
| XY-163 | *Tenacibaculum mesophilum* |
| XY-166-2 | *Mesoflavibacter sabulilitoris* |
| XY-178 | *Tenacibaculum litoreum* |
| XY-185 | *Erythrobacter flavus* |
| XY-203 | *Microbacterium koreense* |
| XY-205 | *Acinetobacter vivianii* |
| XY-207-2 | *Erythrobacter flavus* |
| XY-209 | *Microbacterium aquimaris* |
| XY-210-2 | *Pseudoruegeria aquimaris* |
| XY-221 | *Mesoflavibacter zeaxanthinifaciens* |
| XY-230 | *Erythrobacter flavus* |
| XY-238 | *Erythrobacter flavus* |
| XY-270 | *Pararhodobacter aggregans* |
| XY-322 | *Paracoccus homiensis* |
| XY-336 | *Erythrobacter citreus* |
| XY-337 | *Neptunomonas concharum* |
| XY-339 | *Roseomonas mucosa* |
| XY-355 | *Sphingorhabdus litoris* |
| XY-379 | *Bacillus wiedmannii* |

Table S2. Shown is the classification of all bacterial strains with QQ bioactivity associated with an *Onchidium* sp., an invertebrate collected from Dapeng Bay at Shenzhen.
